# Commonalities and distinctions between the type 2 diabetes mellitus and Alzheimer’s disease: a systematic review and multimodal neuroimaging meta-analysis

**DOI:** 10.3389/fnins.2023.1301778

**Published:** 2023-12-06

**Authors:** Hao Xie, Ying Yu, Yang Yang, Qian Sun, Ze-Yang Li, Min-Hua Ni, Si-Ning Li, Pan Dai, Yan-Yan Cui, Xin-Yu Cao, Nan Jiang, Li-Juan Du, Wen Gao, Jia-Jun Bi, Lin-Feng Yan, Guang-Bin Cui

**Affiliations:** ^1^Department of Radiology, Functional and Molecular Imaging Key Lab of Shaanxi Province, Tangdu Hospital, Fourth Military Medical University (Air Force Medical University), Xi’an, Shaanxi, China; ^2^Faculty of Medical Technology, Xi’an Medical University, Xi’an, Shaanxi, China; ^3^Faculty of Medical Technology, Shaanxi University of Chinese Medicine, Xianyang, Shaanxi, China; ^4^Faculty of Medical Technology, Medical School of Yan’an University, Yan’an, Shaanxi, China; ^5^Student Brigade, Fourth Military Medical University, Xi’an, Shaanxi, China

**Keywords:** functional neuroimaging, Alzheimer’s disease, type 2 diabetes mellitus, functional magnetic resonance imaging, coordinated-based meta-analysis

## Abstract

**Background:**

Alzheimer’s disease (AD) and type 2 diabetes mellitus (T2DM) are aging related diseases with high incidence. Because of the correlation of incidence rate and some possible mechanisms of comorbidity, the two diseases have been studied in combination by many researchers, and even some scholars call AD type 3 diabetes. But the relationship between the two is still controversial.

**Methods:**

This study used seed-based d mapping software to conduct a meta-analysis of the whole brain resting state functional magnetic resonance imaging (rs-fMRI) study, exploring the differences in amplitude low-frequency fluctuation (ALFF) and cerebral blood flow (CBF) between patients (AD or T2DM) and healthy controls (HCs), and searching for neuroimaging evidence that can explain the relationship between the two diseases.

**Results:**

The final study included 22 datasets of ALFF and 22 datasets of CBF. The results of T2DM group showed that ALFF increased in both cerebellum and left inferior temporal gyrus regions, but decreased in left middle occipital gyrus, right inferior occipital gyrus, and left anterior central gyrus regions. In the T2DM group, CBF increased in the right supplementary motor area, while decreased in the middle occipital gyrus and inferior parietal gyrus. The results of the AD group showed that the ALFF increased in the right cerebellum, right hippocampus, and right striatum, while decreased in the precuneus gyrus and right superior temporal gyrus. In the AD group, CBF in the anterior precuneus gyrus and inferior parietal gyrus decreased. Multimodal analysis within a disease showed that ALFF and CBF both decreased in the occipital lobe of the T2DM group and in the precuneus and parietal lobe of the AD group. In addition, there was a common decrease of CBF in the right middle occipital gyrus in both groups.

**Conclusion:**

Based on neuroimaging evidence, we believe that T2DM and AD are two diseases with their respective characteristics of central nervous activity and cerebral perfusion. The changes in CBF between the two diseases partially overlap, which is consistent with their respective clinical characteristics and also indicates a close relationship between them.

**Systematic review registration:**

PROSPERO [CRD42022370014].

## 1 Introduction

Diabetes is a chronic metabolic disease characterized by hyperglycemia (Zheng et al., 2018), and type 2 diabetes mellitus (T2DM) characterized by insulin dysfunction accounts for the majority (about 95%) (Bruno et al., 2005; Holman et al., 2015). It is estimated that the prevalence rate of diabetes will gradually increase from 6.3% in 2019 to 10.2% in 2030, when the population will reach 578 million, becoming a serious global public health problem (Saeedi et al., 2019). The complications of diabetes include retinopathy, renal failure, heart disease, cerebrovascular disease (Kautzky-Willer et al., 2016; Zheng et al., 2018). In addition, the cognitive impairment caused by diabetes is also increasingly concerned. Research reports that about 25–36% of diabetes patients have cognitive impairment (Geijselaers et al., 2015), and progress to dementia more quickly than healthy people (Exalto et al., 2013; Biessels and Despa, 2018), among which the risk of Alzheimer’s disease (AD) increases by about 45–90% (Arvanitakis et al., 2004; Wang et al., 2012).

AD is a chronic neurodegenerative disease with hidden onset (Liu et al., 2019). It is the most common type of dementia, accounting for about 70% (Burns and Iliffe, 2009), and the incidence rate is increasing year by year (Alzheimer’s Association, 2021; Scheltens et al., 2021). The pathological feature of AD is hyperphosphorylated TAU protein deposition in the cells, and forms the neurofibrillary tangles when occurring in the nerve cells (Ballard et al., 2011; Scheltens et al., 2021). According to previous studies, T2DM and AD share many common characteristics, including being highly prevalent age-related diseases with a long prodromal period and being chronic complex diseases (Kubis-Kubiak et al., 2019; Diniz Pereira et al., 2021). In addition, T2DM and AD have many pathological mechanisms in common caused by insulin resistance (Janson et al., 2004), such as metabolic syndrome (Więckowska-Gacek et al., 2021), advanced glycation end products (AGEs) (Byun et al., 2017), insulin signal transduction disorder (De Felice et al., 2022), etc. Therefore, some researchers believe that AD is a late complication with the development of T2DM, which can even be called type 3 diabetes (de la Monte et al., 2018; Nguyen et al., 2020). However, another group of scholars believe that the two diseases are different, and T2DM is only a high-risk factor for AD (Moran et al., 2015), which leads to faster disease progress (Chornenkyy et al., 2019). At present, the relationship between T2DM and AD is still unclear, especially the brain damage caused by the two diseases. However, central insulin resistance and signal transduction abnormalities caused by these two diseases are becoming the mainstream (Diehl et al., 2017; Kellar and Craft, 2020).

In clinical studies, researchers tried to use various methods such as electroencephalogram (EEG), positron emission tomography (PET), single photon emission computed tomography (SPECT) and magnetic resonance imaging (MRI) to clarify the process of brain changes in T2DM or AD (Bucerius et al., 2012; Chen and Zhong, 2013; Brundel et al., 2014; Matsuda, 2016; Benwell et al., 2020). The resting-state functional MRI (rs-fMRI) has been increasingly used because of its non-invasive, efficient, high spatial resolution in detecting central nervous system. Amplitude low-frequency fluctuation (ALFF) and cerebral blood flow (CBF) are more widely used indicator derived from fMRI. ALFF is a measure of resting state blood oxygen level dependent (BOLD) signal changes, reflecting local neural activity (Zou et al., 2008), while CBF measured by arterial spin labeling (ASL) technique which can reflect cerebral perfusion (Williams et al., 1992). ALFF and CBF have always been regarded as two independent indicators, but studies have confirmed that CBF was involved in regulating the change of BOLD signal (Kannurpatti et al., 2008; Tak et al., 2014), and they can reflect the intensity of local neural activity in direct and indirect ways (Kim and Lee, 2004; Yu-Feng et al., 2007), respectively. Furthermore, these two indicators can be combined for analysis to represent the neurovascular coupling status of brain regions (Hu et al., 2019; Yu et al., 2019). Therefore, it is necessary to conduct research and analysis on these two indicators.

Since the application of fMRI technology, a lot of scientific achievements have been published on ALFF and CBF alteration in T2DM or AD. However, differences in sample size, demographic information, image acquisition techniques and analysis methods among different studies lead to heterogeneity of results. Meta-analysis has emerged to identify abnormal brain activity from a large number of studies. For example, a published meta-analysis in T2DM patients showed a decrease of ALFF in the parietal lobe, occipital lobe, and cingulate gyrus (Macpherson et al., 2017). The meta-analysis in AD patients showed a general decrease of CBF in whole brain, especially in the posterior cingulate gyrus and temporal parietal lobe (Zhang et al., 2021), while the meta-analysis in T2DM patients showed that CBF decreased in bilateral occipital lobe but increased in right prefrontal lobe and supplementary motor area (Liu et al., 2022). Due to differences of literature inclusion criteria and specific analysis methods, the level of evidence from the combined analysis of the above two indicators might decrease. In summary, it is essential to combine ALFF and CBF for further analysis by using neurovascular coupling coefficient, in order to explore the alteration of brain neural activity in T2DM and AD, and to analyze the similarities and differences of brain damage caused by the two diseases.

The aim of this study is, to perform a voxel-based meta-analysis of ALFF and CBF changes in patients with T2DM and AD, by taking advantage of the large number of whole-brain rs-fMRI studies published in recent years, and to explore whether there are similarities in brain alterations in the two diseases. This is not only helpful to understand the pathophysiology of T2DM and AD more accurately, but also can provide evidence of brain damages from the perspective of imaging, which is helpful to reveal the pathogenesis and to find the promising biomarkers.

## 2 Methods

### 2.1 Protocol and guidance

The meta-analysis was conducted in accordance with the guidelines of the Preferred Reporting Items for Systematic Reviews and Meta-Analyses (PRISMA) and 10 simple rules for neuroimaging meta-analysis (Müller et al., 2018; Page et al., 2021). The protocol of this neuroimaging meta-analysis was registered on PROSPERO (CRD42022370014).^1^

### 2.2 Search strategy

We used a systematic search strategy to identify published relevant studies in databases including PubMed, Web of Science, from Jan 1, 2007 to Sep 1, 2022. Divided the search process into two parts based on the type of indicator. The first part used keywords (“Diabetes Mellitus, Type 2” OR “Type 2 Diabetes” OR “Diabetes Mellitus, Type II” OR “NIDDM” OR “T2DM” OR “Alzheimer Disease” OR “Alzheimer*” OR “dement*” OR “AD”) AND (“amplitude of low frequency fluctuation” OR “ALFF” OR “low frequency fluctuation” OR “LFF” OR “amplitude of low frequency oscillation” OR “LFO”). The second part used keywords (“Diabetes Mellitus, Type 2” OR “Type 2 Diabetes” OR “Diabetes Mellitus, Type II” OR “NIDDM” OR “T2DM” OR “Alzheimer Disease” OR “Alzheimer*” OR “dement*” OR “AD”) AND (“Cerebrovascular Circulation” OR “arterial spin labeling” OR “ASL” OR “Cerebral Blood Flow” OR “CBF”).

### 2.3 Study selection

After completing the search, duplicate studies were first excluded. When extracting information in the study by reading the full text, if there was important information that could not be found, such as coordinate values, non-online manuscripts, etc., we contacted the corresponding author by email. After information extraction, studies conforming to the research will be included in the following: (1) an article was published, rather than the abstract, lecture or letters; (2) assessed CBF or ALFF in whole brain analysis; (3) participants were classified into healthy controls (HCs) and T2DM and/or AD groups in cross-sectional and at the baseline of longitudinal studies; (4) the article clearly reported peak coordinates in stereotactic three-dimensional coordinates (MNI or Talairach); (5) be able to extract the t value, z values or *p* values; and (6) subjects were adults (18–75 years old). Exclusion criteria will be: (1) the study participants were individuals diagnosed with dementia other than AD; (2) other neuropsychiatric disorders, macrovascular complications, craniocerebral trauma, and inflammatory lesions of the central nervous system; (3) no HCs; (4) not related to ALFF and CBF; (5) studies with ROI analysis; (6) research on minors; (7) secondary study; and (8) neuroimaging quality score<16 or JBI score<12.

### 2.4 Quality assessment

We referred to the previous high-quality literature and used the methodological assessment checklist which was specific for neuroimaging meta-analysis to evaluate the quality of the included study (Pan et al., 2017; Supplementary Figure S1; Supplementary Tables S4, S5). In addition, only cross-sectional information was extracted after the study was included, so we introduced the Joanna Briggs Institute (JBI) critical appraisal checklist of the cross-sectional study for secondary assessment (Ma et al., 2020; Supplementary Figure S2; Supplementary Table S6). The quality of the study was first independently evaluated by two reviewers (H.X and ZY.L, Radiologist), and the consistent evaluation results would be adopted. If there were differences in the evaluation results, the third reviewer (LF.Y, Deputy Chief Radiologist and Associate Professor) would evaluate and make final decision.

### 2.5 Voxel-wise meta-analysis of CBF and ALFF abnormalities

The meta-analyses of ALFF and CBF were performed in the “Gray Matter” templates of the anisotropic effect size-signed differential mapping (AES-SDM) (Radua and Mataix-Cols, 2009; Radua et al., 2012; Radua and Mataix-Cols, 2012; Radua et al., 2014), which has been widely used in the meta-analysis of neuroimaging (Barona et al., 2019; Li et al., 2022). The specific research process has been reported in detail in previous studies (Radua and Mataix-Cols, 2009; Ferreira and Busatto, 2010; Radua et al., 2012), so we summarized the methods as follows: First, extracting the effective coordinates of CBF or ALFF abnormalities (increased or decreased) between T2DM patients or AD patients and HCs in each data set and the size of their brain impacts, and using various heterotypic Gaussian kernels to reconstruct the statistical map on MNI coordinates. Then, the study combined the random effect model considering sample size, intra-study variability and between-study heterogeneity to generate a mean map. Finally, MRICRON^2^ software were used to visualize the data.

According to the research of software developers, we have adopted the recommended settings (FWHM = 20 mm, *p* = 0.005, peak height *Z* = 1, and cluster extent ≥20 voxels) in this study (Radua et al., 2012). When extracting data from research, if only z value or *p* value was provided, it can be analyzed by converting it to *t* value through https://www.sdmproject.com/utilities/?show=Statistics. According to the software instruction, the following five standard steps will be followed when processing data: (1) Global analysis, (2) Pre-processing, (3) Mean analysis, (4) Threshold analysis, and (5) Extract peak coordinates and Bias Test.

Next, we compared the covariant brain regions (increased or decreased) of ALFF and CBF in T2DM compared with HCs through quantitative meta-analysis of brain regions with differences among groups obtained from previous analysis, and used standard randomization test to determine statistical significance. The same analysis was performed for AD group. In this process, we took demographic information with statistical differences as covariates. In addition, by combining the result graph of threshold element analysis, we studied the increase/decrease overlap of ALFF between T2DM and AD, and compared the voxel number and z value in the actual interaction area and visualized results. Similarly, we conducted the same analysis on covariant of CBF between T2DM and AD.

### 2.6 Heterogeneity, sensitivity and publication bias

Extract the MNI peak coordinates with statistical differences, and obtain the standard heterogeneity test I^2^. If *I*^2^ ≥ 50%, it means significant heterogeneity (Egger et al., 1997). Funnel plots were used to test whether there was publication bias. Asymmetric funnels or *p* < 0.05 were considered to have publication bias (Sterne et al., 2011). These analyses were performed using the SDM-PSI version 6.21.^3^ Finally, jackknife sensitivity was used for sensitivity analysis of whole brain voxels. The specific method was to check the stability of results by repeating the same analysis process after excluding one data set each time (Radua et al., 2014). This procedure aimed to analyze the repeatability of the results. If a result was significant different in all or most (>50%) of the study combinations, we believed that the result was highly replicable (Radua and Mataix-Cols, 2009).

### 2.7 Meta-regression analysis

In the study, the linear regression in AES-SDM was used for meta-regression to explore the impact of demographic information and clinical variables such as years of education, course of disease, and clinical evaluation scale scores on the results. Regression analysis could exclude the regions outside the brain obtained from principal component analysis (Yao et al., 2021).

## 3 Results

### 3.1 Included studies

**Figure 1:**
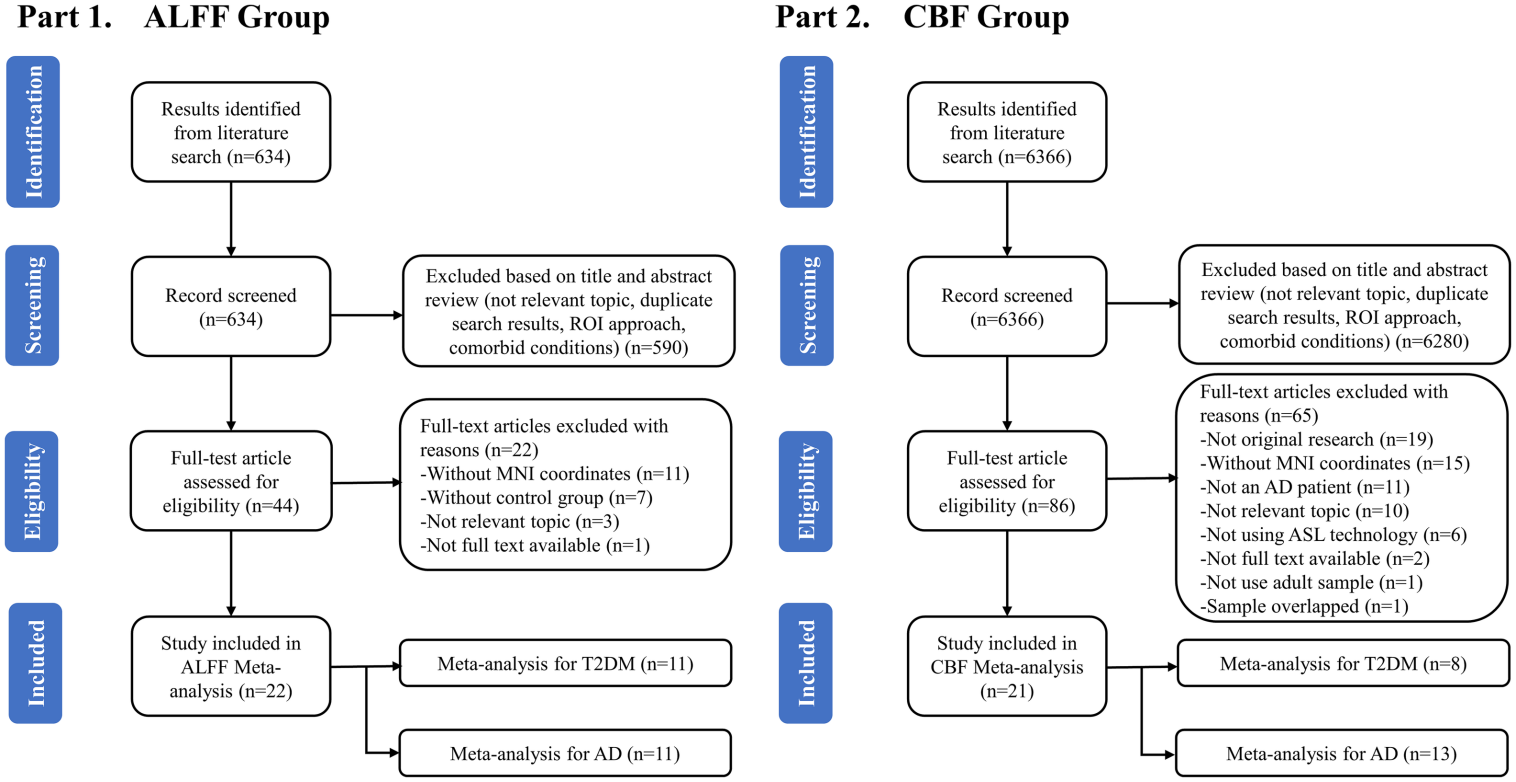
Flowchart for identifying studies to be included in the meta-analysis.

A total of 634 studies were obtained from the first part of the search. After preliminary removing the duplicates and reviewing the titles and abstracts, 44 studies were retained and considered potentially eligible for inclusion. Then, after a detailed reading of the full article text, another 22 studies were excluded. Finally, 22 studies including 22 data sets met the criteria and were included to analyze the ALFF differences between T2DM and AD patients, including 11 studies on AD patients and 11 studies on T2DM patients (Figure 1). A total of 6,366 studies were obtained from the second part of the search. After preliminary removing the duplicates and reviewing the titles and abstracts, 86 studies were retained and considered potentially eligible for inclusion. Then, after a detailed reading of the full article text, another 65 studies were excluded. Finally, 21 studies including 21 data sets met the criteria and were included to analyze the CBF differences between T2DM and AD patients, including 13 studies on AD patients and 8 studies on T2DM patients. A total of 43 studies were included for this meta-analysis (Figure 1).

### 3.2 Sample characteristics

#### 3.2.1 T2DM

In all of T2DM studies included in ALFF analysis, 302 patients with T2DM (171 males and 131 females, mean age = 56.00 years) and 302 HCs (153 males and 149 females, mean age = 55.36 years) were included (Detailed demographic and clinical information is shown in Table 1, and radiological parameters are shown in Supplementary Tables S2, S3). There was no significant difference in gender (*χ^2^* = 2.157, *p* = 0.14) and age (standardized mean difference [SMD] = 0.11; 95% confidence interval [CI] = [−0.05, 0.27], *Z* = 1.30, *p* = 0.19) distribution between the two groups. Among all included CBF related studies, 286 T2DM patients (150 males and 136 females) and 280 HCs (131 males and 149 females) were included. No significant difference was observed between patients with T2DM and HCs in gender (*χ^2^* = 1.814, *p* = 0.18) and age (SMD = 0.91; CI = [−0.34, 2.16], *Z* = 1.43, *p* = 0.15) distribution.

**Table 1:** Demographic, clinical and cognitive characteristics of T2DM patients and HCs included in the meta-analysis.

Study	Indicator	Subjects (male/female)		Mean age (SD)		Education years (SD)		Duration years (SD)	HbA1c (%) (SD)		MMSE (SD)		MOCA (SD)	
		T2DM	HC	T2DM	HC	T2DM	HC		T2DM	HC	T2DM	HC	T2DM	HC
Xia et al. (Zheng et al., 2018)	ALFF	28 (15/13)	29 (13/16)	58.7 (8.1)	57.7 (7.2)	9.9 (3.7)	11.0 (2.0)	9.8 (5.5)	7.9 (1.7)	5.6 (0.4)	/	/	23.2 (3.1)	24.1 (2.6)
Chen et al. (Bruno et al., 2005)	ALFF	18 (8/10)	18 (7/11)	61.7 (7.6)	62.1 (11.0)	/	/	13.8 (7.9)	7.3 (1.1)	/	26.1 (2.2)	26.6 (2.0)	/	/
Cui et al. (Holman et al., 2015)	ALFF	29 (14/15)	27 (11/16)	58.3 (7.3)	57.8 (5.9)	10.4 (4.0)	10.2 (2.5)	9.3 (3.8)	7.9 (1.7)	5.6 (0.4)	28.3 (1.4)	29.0 (1.1)	23.6 (2.9)	27.3 (1.1)
Wang et al. (Saeedi et al., 2019)	ALFF	26 (17/9)	26 (17/9)	54.7 (10.4)	54.9 (9.8)	11.2 (3.8)	10.7 (3.2)	7^a^	8.3 (1.4)	/	27.8 (2.5)	28.3 (1.3)	24^a^	26.5^a^
Zhou et al. (Kautzky-Willer et al., 2016)	ALFF	14 (6/8)	17 (10/7)	63.5 (6.9)	63.8 (5.8)	10.6 (2.7)	11.7 (3.0)	6.5 (2.1)	7.8 (1.0)	5.4 (0.6)	25.1 (2.0)	28.6 (1.1)	/	/
Wang et al. (Geijselaers et al., 2015)	ALFF	21 (10/11)	16 (7/9)	54.9 (9.9)	54.8 (5.7)	/	/	9.5 (5.0)	8.4 (1.7)	5.6 (0.9)	28.2 (1.1)	29.0 (0.7)	21.7 (0.7)	25.2 (1.9)
Yu et al. (Biessels and Despa, 2018)	ALFF, CBF	33 (28/5)	33 (22/11)	53.5 (8.4)	51.0 (5.3)	12.8 (2.4)	12.9 (3.5)	7.1 (5.2)	8.1 (1.7)	5.6 (0.3)	28.9 (0.9)	28.5 (1.1)	26.5 (2.1)	26.8 (2.0)
Liu et al. (Exalto et al., 2013)	ALFF	37 (24/13)	37 (17/20)	57.6 (7.1)	57.9 (5.7)	11.6 (3.9)	10.9 (2.3)	8.7 (5.5)	7.6 (1.5)	5.7 (0.4)	28.0 (1.5)	28.5 (1.2)	22.5 (2.7)	24.2 (2.7)
Shi et al. (Arvanitakis et al., 2004)	ALFF	31 (16/15)	31 (16/15)	56.0 (4.6)	56.5 (4.3)	/	/	/	/	/	/	/	/	/
Li et al. (Wang et al., 2012)	ALFF	30 (15/15)	30 (15/15)	49.2 (5.5)	45.8 (6.4)	12 (6, 16)^b^	9 (6, 16)^b^	/	8.7 (2.2)	/	/	/	26.5 (26, 29)^b^	28.5 (26, 30)^b^
Qi et al. (Liu et al., 2019)	ALFF	35 (18/17)	38 (18/20)	54.2 (8.7)	53.5 (7.7)	/	/	9.9 (5.1)	7.5 (1.3)	5.6 (0.4)	/	/	/	/
Xia et al. (Burns and Iliffe, 2009)	CBF	38 (17/21)	40 (21/19)	56.0 (6.1)	57.1 (7.6)	9.6 (3.0)	10.3 (1.9)	7.1 (3.5)	7.2 (1.1)	5.6 (0.3)	29.0 (0.9)	29.1 (1.0)	/	/
Jansen et al. (Alzheimer’s Association, 2021)	CBF	41 (23/18)	39 (22/17)	61.1 (9.6)	62.6 (6.6)	/	/	9.8 (6.7)	6.7 (0.4)	5.6 (0.4)	28.6 (1.4)	29.4 (0.8)	/	/
Cui et al. (Scheltens et al., 2021)	CBF	40 (21/19)	41 (13/28)	60.5 (6.9)	57.9 (6.5)	10.0 (3.4)	10.3 (2.3)	8.9 (5.0)	7.7 (1.6)	5.6 (0.3)	28.3 (1.0)	28.6 (1.2)	/	/
Dai et al. (Ballard et al., 2011)	CBF	41 (19/22)	32 (16/16)	65.5 (8.3)	67.3 (10.1)	15.4 (3.8)	16.1 (3.0)	9.9 (7.9)	7.3 (1.3)	5.7 (0.3)	28.6 (1.5)	28.9 (1.6)	/	/
Shen et al. (Diniz Pereira et al., 2021)	CBF	36 (17/19)	36 (14/22)	57.6 (6.2)	56.2 (6.8)	9.1 (1.5)	9.8 (2.9)	5.4 (4.9)	/	/	/	/	25.7 (0.9)	26.0 (0.8)
Zhang et al. (Kubis-Kubiak et al., 2019)	CBF	26 (10/16)	26 (11/15)	51.9 (10.7)	48.2 (6.7)	10.3 (3.7)	11.6 (4.5)	9.2 (7.1)	/	/	26.9 (3.9)	27.7 (2.3)	23.5 (5.6)	25.0 (2.9)
Huang et al. (Janson et al., 2004)	CBF	31 (15/16)	33 (12/21)	53.4 (9.1)	51.6 (9.8)	/	/	/	7.3 (1.4)	/	/	/	/	/

Data are presented as mean (SD), or range. T2DM, type 2 diabetes mellitus; HC, healthy control; SD, standard deviation; HbA1c, glycosylated hemoglobin A1c; MMSE, mini-mental state examination; MoCA, montreal cognitive assessment.

^a^ Only the mean value of the data is given in the article.

^b^ Mean (range).

/means no relevant information was provided in the study.

#### 3.2.2 AD

In all of AD studies included in ALFF analysis, 390 patients with AD (160 males and 230 females, mean age = 69.23 years) and 492 HCs (183 males and 309 females, mean age = 68.93 years) were included (Detailed demographic and clinical information is shown in Table 2, and radiological parameters are shown in Supplementary Tables S2, S3). No significant difference were observed between the two groups in gender (*χ^2^* = 1.343, *p* = 0.25), but there was significant difference in age distribution (SMD = 2.74; CI = [1.56, 3.92], *Z* = 4.56, *p* < 0.00001). Among all included CBF related studies, 310 AD patients (121 males and 189 females) and 335 HCs (142 males and 193 females) were included. No significant difference were observed between AD patients and HCs in gender (*χ^2^* = 0.751, *p* = 0.39), while there was significant difference in age (SMD = 1.73; CI = [0.90, 2.56], *Z* = 4.10, *p* < 0.0001).

**Table 2:** Demographic, clinical and cognitive characteristics of AD patients and HCs included in the meta-analysis.

Study	Indicator	Subjects (male/female)		Mean age (SD)		Education years (SD)		MMSE (SD)		MOCA (SD)		CDR scale	
		AD	HC	AD	HC	AD	HC	AD	HC	AD	HC	AD	HC
Wang et al. (Więckowska-Gacek et al., 2021)	ALFF	16 (8/8)	22 (7/15)	69.6 (7.7)	66.6 (7.7)	10.1 (3.4)	10.0 (3.9)	18.5 (3.2)	28.6 (0.6)	/	/	1.0 (0.0)	0
Xi et al. (Byun et al., 2017)	ALFF	20 (9/11)	20 (10/10)	68.8 (8.7)	64.7 (5.6)	12.1 (4.4)	12.2 (2.5)	20.6 (2.3)	28.2 (1.8)	/	/	1.0 (0.0)	0
Veldsman et al. (De Felice et al., 2022)	ALFF	44 (22/22)	128 (40/88)	78.0 (8.7)	74.6 (6.1)	13.1 (4.8)	13.5 (8.9)	/	/	/	/	/	/
Zheng et al. (de la Monte et al., 2018)	ALFF	14 (6/8)	14 (6/8)	66.9 (8.9)	66.7 (5.8)	8.7 (3.0)	11.8 (4.1)	16.3 (4.9)	28.1 (1.3)	13.3 (4.9)	27.4 (1.9)	(1.0, 2.0)^a^	0
Li et al. (Nguyen et al., 2020)	ALFF	16 (7/9)	69 (23/46)	74.7 (8.5)	74.8 (6.6)	16.4 (2.6)	16.4 (2.4)	20.8 (4.4)	29.1 (1.0)	/	/	1.0 (0.6)	0
Zeng et al. (Moran et al., 2015)	ALFF	14 (9/5)	11 (0/11)	75.5 (4.1)	75.4 (8.2)	15.6 (2.9)	16.1 (6.4)	21.4 (3.7)	29.5 (1.0)	/	/	/	0
Zheng et al. (Chornenkyy et al., 2019)	ALFF, CBF	40 (18/22)	30 (15/15)	65.0 (10.0)	64.0 (8.0)	11.2 (3.2)	12.6 (4.6)	14.0 (6.0)	28.0 (2.0)	14.9 (3.2)	28.6 (0.7)	(0.5, 2.0)^a^	0
Yang et al. (Kellar and Craft, 2020)	ALFF	44 (15/29)	55 (22/33)	71.0 (10.0)	63.4 (8.0)	9.0 (5.9)	11.0 (5.0)	16.5 (6.4)	28.1 (2.1)	12.6 (5.3)	26.1 (3.2)	(1.0, 2.0)^a^	0
Li et al. (Diehl et al., 2017)	ALFF	111 (37/74)	73 (32/41)	68.3 (9.4)	66.3 (9.5)	7.9 (4.4)	8.3 (3.4)	17.2 (5.6)	28.8 (0.3)	13.4 (6.3)	27.2 (1.7)	/	0
Chen et al. (Brundel et al., 2014)	ALFF	31 (12/19)	50 (18/32)	69.9 (11.0)	64.5 (4.4)	8.2 (4.6)	10.5 (2.7)	12.0 (4.5)	27.2 (1.8)	/	/	(1.0, 2.0)^a^	0
Zhan et al. (Chen and Zhong, 2013)	ALFF	40 (17/23)	20 (10/10)	60.5 (7.4)	61.0 (7.3)	9.7 (4.8)	9.9 (4.9)	17.5 (5.5)	27.0 (4.0)	/	/	/	
Asllani et al. (Benwell et al., 2020)	CBF	12 (7/5)	20 (8/12)	70.7 (8.7)	72.4 (6.5)	14.5 (3.8)	15.8 (2.3)	38.7 (11.1)^b^	53.5 (2.8)^b^	/	/	1.0 (0.0)	0
Dai et al. (Bucerius et al., 2012)	CBF	37 (13/24)	41 (14/27)	83.6 (3.5)	82.1 (3.6)	/	/	85.1 (9.4)^c^	95.0 (4.5)^c^	/	/	(1.0, 2.0)^a^	0
Yoshiura et al. (Matsuda, 2016)	CBF	20 (10/10)	23 (11/12)	73.5 (9.6)	72.9 (6.7)	/	/	20.4 (4.3)	29.3 (0.9)	/	/	/	/
Chao et al. (Zou et al., 2008)	CBF	13 (3/10)	35 (5/30)	77.1 (5.0)	76.0 (7.8)	16.7 (2.9)	16.5 (2.8)	27.5 (1.8)	28.5 (1.7)	/	/	(0.5, 1)^a^	0
Dashjamts et al. (Williams et al., 1992)	CBF	23 (9/14)	23 (11/12)	74.6 (8.9)	73.2 (6.9)	/	/	21.1 (4.4)	29.4 (0.9)	/	/	/	/
Alexopoulos et al. (Kannurpatti et al., 2008)	CBF	19 (11/8)	24 (8/16)	72.0 (9.4)	67.1 (6.1)	/	/	/	/	/	/	/	/
Mak et al. (Tak et al., 2014)	CBF	13 (3/10)	15 (1/14)	75.4 (6.8)	70.8 (6.0)	/	/	16.3 (4.6)	28.5 (2.0)	/	/	/	/
Kim et al. (Yu-Feng et al., 2007)	CBF	25 (4/21)	25 (9/16)	70.9 (9.8)	68.4 (5.6)	/	/	17.2 (4.8)	27.3 (2.8)	/	/	(0.5, 2.0)^a^	0
Ding et al. (Kim and Lee, 2004)	CBF	24 (5/19)	21 (8/13)	74.6 (6.7)	69.6 (5.9)	11.6 (4.2)	12.1 (3.4)	16.0 (3.9)	29.4 (1.0)	/	/	2.1 (0.7)	0
Roquet et al. (Hu et al., 2019)	CBF	25 (8/17)	21 (9/12)	73.6 (9.1)	64.8 (8.6)	/	/	19.5 (3.4)	28.9 (1.0)	/	/	/	/
Duan et al. (Yu et al., 2019)	CBF	40 (12/28)	58 (27/31)	84.1 (3.5)	83.4 (3.7)	13.3 (2.9)	14.6 (2.8)	83.6 (10.0)^c^	95.0 (3.9)^c^	/	/	/	/
Soman et al. (Macpherson et al., 2017)	CBF	19 (11/8)	21 (11/10)	66.7 (5.3)	64.6 (5.7)	/	/	/	/	/	/	(0.5, 1.5)^a^	0

Data are presented as mean (SD), or range. AD, Alzheimer’s disease; HC, healthy control; SD, standard deviation; MMSE, mini-mental state examination; MoCA, montreal cognitive assessment; CDR, clinical dementia rating.

^a^ The study did not give the mean value and variance, only the range.

^b^ MMMS, Modified Mini-Mental Status Examination score.

^c^ 3MSE, Modified Mini-Mental State Examination score.

/means no relevant information was provided in the study.

### 3.3 ALFF meta-analysis

#### 3.3.1 T2DM *vs.* HCs

**Figure 2:**
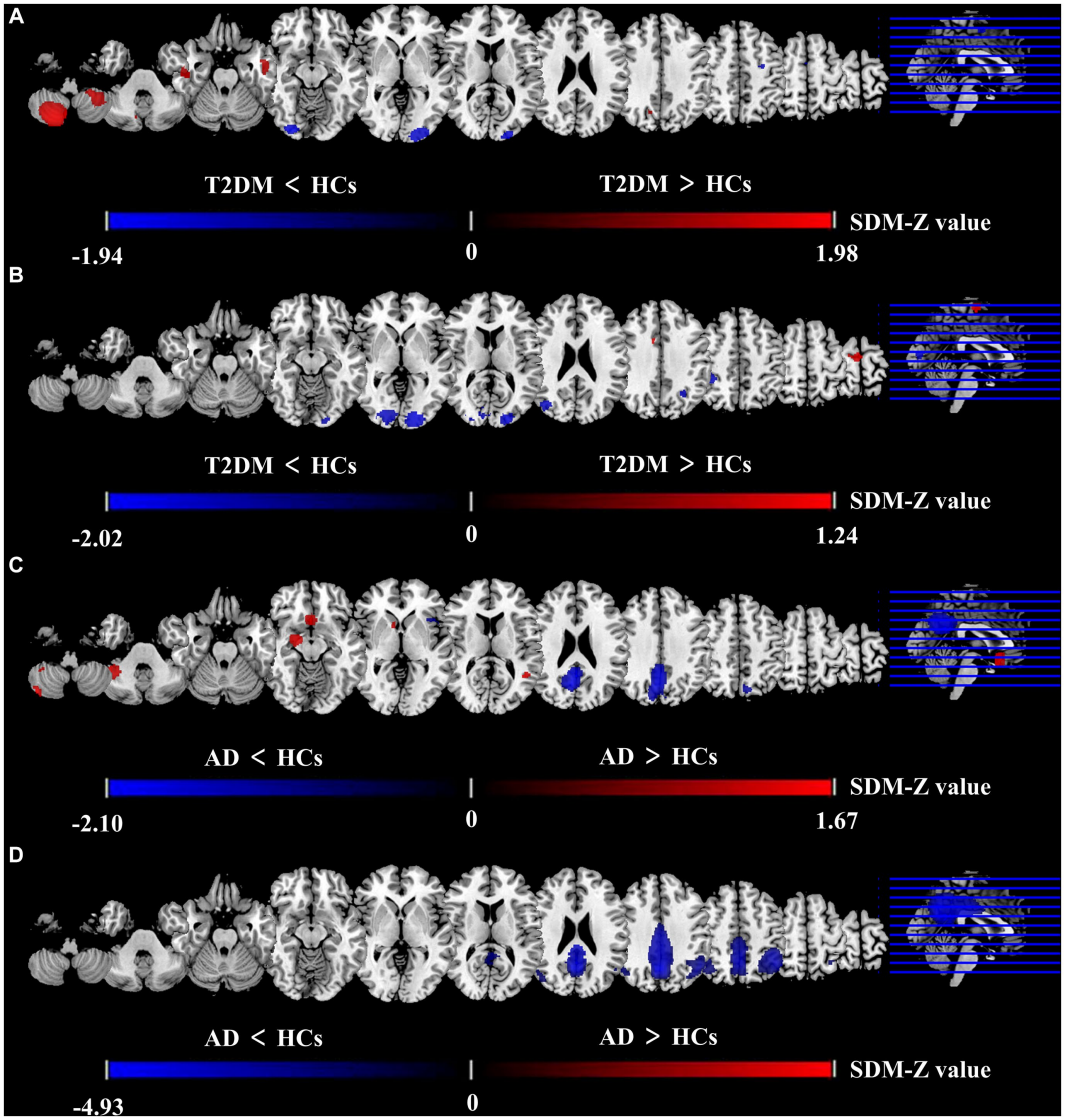
Differences between two groups in CBF and ALFF in meta-analysis results. Results of the meta-analysis **(A)** ALFF difference between T2DM and HCs. **(B)** CBF difference between T2DM and HCs. **(C)** ALFF difference between AD and HCs. **(D)** CBF difference between AD and HCs.

The brain map derived from meta-analysis showed that compared to HCs, ALFF in the T2DM group increased in the cerebellum (CER) and left inferior temporal gyrus (ITG. L), while decreased in the left middle occipital gyrus (MOG. L), right inferior occipital gyrus (IOG. R), and left precentral gyrus (preCG. L) (Figure 2). These regions existed significant heterogeneity (*I*^2^ > 50%), so random effect model was selected for analysis. Except for preCG.L, there was no publication bias in other brain regions. The research of Zhou et al. (2014) led to publication bias in preCG.L. Jackknife sensitivity analysis showed that the above brain regions were highly repeatable and the results were reliable (Supplementary Table S7).

#### 3.3.2 AD *vs.* HCs

The meta-analysis brain maps showed that compared to HC, ALFF in the AD group increased in CER. R, right striatum, and right hippocampus (HIP. R), while decreased in the precuneus gyrus (PCUN) and right superior temporal gyrus (STG. R) (Figure 2). These regions existed significant heterogeneity (*I*^2^ > 50%), so random effect model was selected for analysis. There was no publication bias in all brain regions. Jackknife sensitivity analysis showed that the above brain regions were highly repeatable and the results were reliable (Supplementary Table S8).

#### 3.3.3 (T2DM *vs.* HCs) and (AD *vs.* HCs) combined analysis

The results of a joint two parts analysis showed that compared to HCs, T2DM and AD did not have brain regions where ALFF increased or decreased simultaneously.

### 3.4 CBF meta-analysis

#### 3.4.1 T2DM *vs.* HCs

The meta-analysis brain maps showed that compared to HCs, the T2DM group had an increase of CBF in the right supplementary motor area (SMA. R), while a decrease of CBF in the middle occipital gyrus (MOG) and inferior parietal gyri (IPG) (Figure 2). These regions existed significant heterogeneity (*I*^2^ > 50%), so random effect model was selected for analysis. There was no publication bias in all brain regions. Jackknife sensitivity analysis indicated that the most reliable data had been obtained in the above brain regions (Supplementary Table S9).

#### 3.4.2 AD *vs.* HCs

The brain maps showed that in CBF meta-analysis, compared to HC, the AD group’s CBF decreased in PCUN and IPG (Figure 2). These regions existed significant heterogeneity (*I*^2^ > 50%), so random effect model was selected for analysis. There was no publication bias in all brain regions. Jackknife sensitivity analysis indicated that the most reliable data had been obtained in the above brain regions (Supplementary Table S10).

#### 3.4.3 (T2DM vs. HCs) and (AD vs. HCs) combined analysis

**Figure 3:**
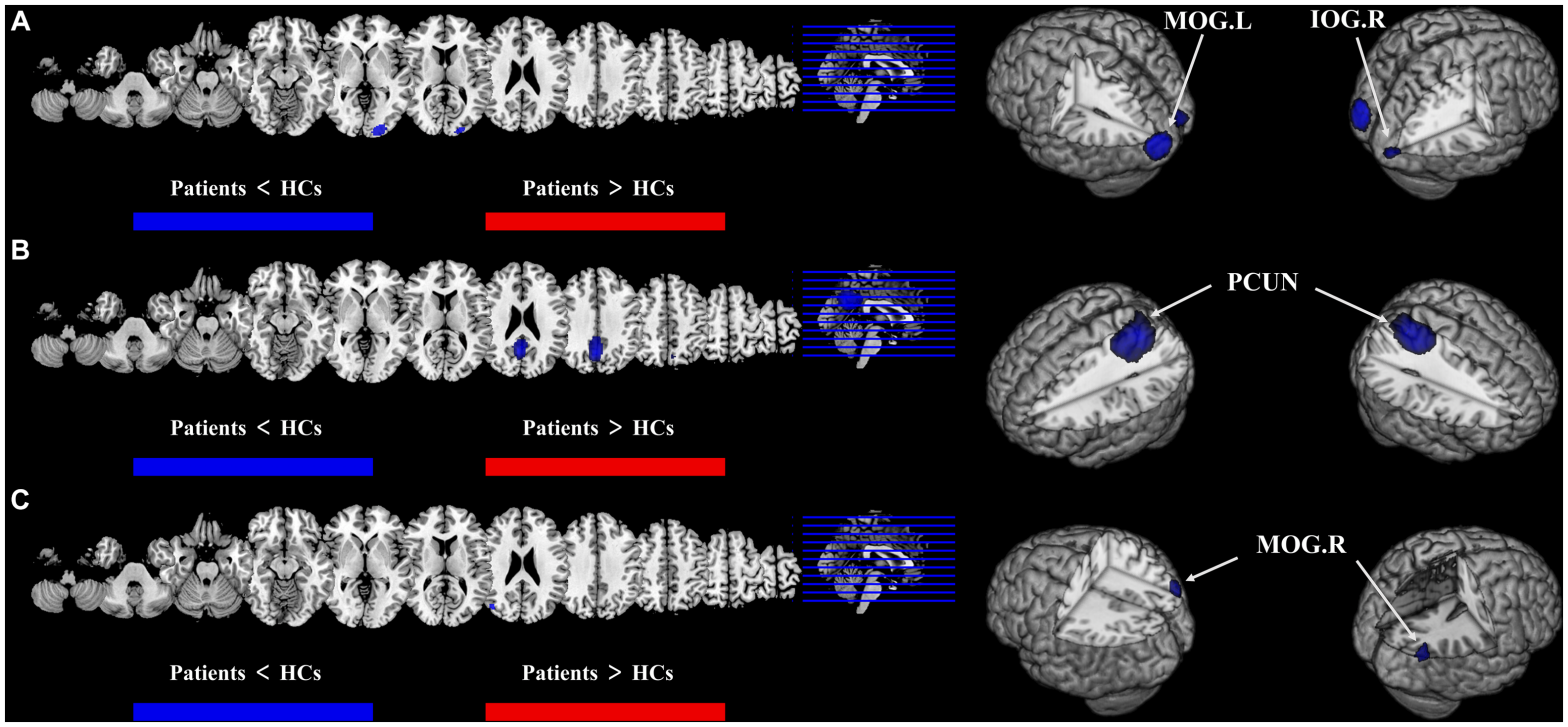
Results of multimodal analysis within a disease and joint analysis between diseases. Results of the meta-analysis **(A)** both ALFF and CBF decreased in T2DM. **(B)** Both ALFF and CBF decreased in AD. **(C)** CBF reduction in both T2DM and AD.

**Figure 4:**
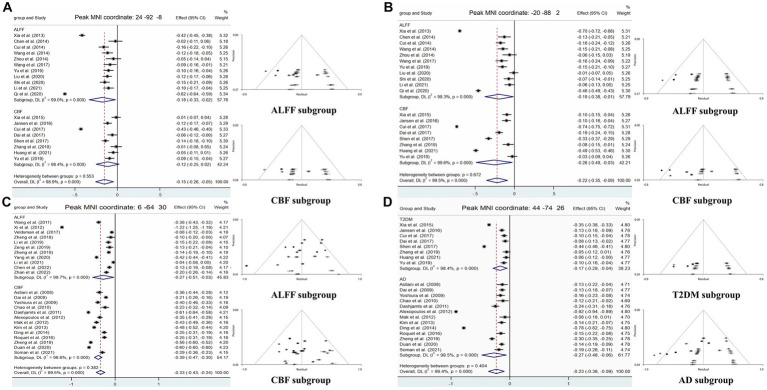
Forest and funnel plots of peak MNI coordinates. Peak MNI coordinate regarding **(A,B)** both ALFF and CBF decreased in T2DM and subgroup results. **(C)** Both ALFF and CBF decreased in AD and subgroup results. **(D)** CBF reduction in both T2DM and AD and subgroup results.

Compared with HCs, CBF of both T2DM patients and AD patients decreased in the MOG.R (peak MNI coordinate: 44, −74, 26, *Z* = −3.059, 56 voxels) (Figure 3). The subgroup analysis of T2DM and AD in this brain region showed significant heterogeneity (*I*^2^ > 50%), so a random effect model was used for analysis. In subgroup analysis, there was no publication bias in this brain region (Figure 4 and Table 3).

**Table 3:** Results of multimodal analysis within a disease and combined analysis between diseases.

Local maximum region	Peak MNI coordinate (x, y, z)	Peak intensity	SDM Z value	Cluster (NO. of voxels)	Breakdown (No. of voxels)	Egger’s test (*p* value)	Heterogeneity (I^2^)	Jackknife sensitivity
Both ALFF and CBF decreased in T2DM								
Right occipital lobe	24, −92, −8	−2.4866	−1.560 (ALFF), −1.403 (CBF)	53	Right occipital lobe (53)	0.545 (ALFF) 0.614 (CBF)	99% (ALFF) 98.4% (CBF)	10/11 (ALFF) 8/8 (CBF)
Left occipital lobe	−20, −88, 2	−2.9759	−1.579 (ALFF), −1.544 (CBF)	564	Right middle occipital gyrus (247)	0.887 (ALFF) 0.833 (CBF)	99.3% (ALFF) 99.6% (CBF)	10/11 (ALFF) 8/8 (CBF)
					Right inferior occipital gyrus (74)			
					Right lingual gyrus (50)			
Both ALFF and CBF decreased in AD								
Precuneus and parietal lobe	6, −64, 30	−9.5112	−2.029 (ALFF), −4.686 (CBF)	1,692	Right precuneus (678)	0.339 (ALFF) 0.640 (CBF)	99.7% (ALFF) 98.6% (CBF)	11/11 (ALFF) 13/13 (CBF)
					Left precuneus (504)			
					Left posterior cingulate gyrus (150)			
					Right median cingulate / paracingulate gyri (108)			
					Right posterior cingulate gyrus (103)			
Both T2DM and AD decreased with CBF								
Right occipital lobe	44, −74, 26	−3.0589	−1.551 (T2DM), −1.971 (AD)	56	Right middle occipital gyrus (48)	0.754 (T2DM) 0.745 (AD)	98.4% (T2DM) 99.5% (AD)	8/8 (T2DM) 13/13 (AD)

T2DM, type 2 diabetes mellitus; AD, Alzheimer’s disease; ALFF, amplitude of low frequency fluctuation; CBF, cerebral blood flow.

### 3.5 Multimodal meta-analysis results

For T2DM, there were two brain regions where CBF and ALFF decreased together, respectively in right occipital lobe (peak MNI coordinate: 24, −92, −8, *Z* = −2.487, 53 voxels) and left occipital lobe (peak MNI coordinate: −20, −88, 2, *Z* = −2.976, 564 voxels) (Figure 3). For AD group, there was a brain region where ALFF and CBF decreased together in bilateral precuneus and parietal lobes (peak MNI coordinate: 6, −64, 30, *Z* = −9.511, 1,692 voxels) (Figure 3). The subgroup analysis of ALFF and CBF in these brain regions showed significant heterogeneity (*I*^2^ > 50%), so a random effect model was used for analysis. In subgroup analysis, there were no publication bias in these brain regions (Figure 4 and Table 3).

### 3.6 Meta-regression

In the regression analysis, we excluded the abnormal brain regions outside the main results. Meta regression analysis showed that general demographic statistics (age and gender) had no significant impact on the main results in both T2DM and AD patients, even if there was a difference in the age of patients of AD. However, in T2DM patients, lower MMSE scores were associated with lower ALFF in the left frontal lobe (peak MNI coordinate: −30, −4, 56, *Z* = 6.432, 115 voxels) and lower CBF in the right parietal lobe (peak MNI coordinate: 38, −36, 44, *Z* = 4.045, 41 voxels).

## 4 Discussion

In this paper, we conducted a multimodal voxel based meta-analysis of T2DM and AD, and obtained the following results: (1) In T2DM patients, ALFF in the CER and ITG.L as well as CBF in the SMA.R increased, while ALFF in the MOG.L, IOG.R, and preCG.L as well as CBF in the MOG and IPL decreased. (2) In AD patients, ALFF in the CER.R, right striatum and HIP.R increased, while ALFF in the PCUN and STG.R as well as CBF in the PCUN and IPG decreased. (3) During multimodal analysis of ALFF and CBF, it was found that in T2DM patients, there was a simultaneous decrease of neural activity and blood perfusion in the area of both occipital lobes, while in AD patients, there was a simultaneous decrease of neural activity and blood perfusion in the parietal lobe. Except for decreased CBF in MOG. R in both type of patients, there were no common changes in other brain regions between the two diseases. (4) Regression analysis showed that general demographic information had no impact on the main results of the meta-analysis, while the MMSE scores of T2DM had an impact on ALFF in the left frontal lobe and CBF in the right parietal lobe.

The results of this meta-analysis showed that both ALFF and CBF of T2DM in occipital region were significantly reduced. As a key area of the visual cortex, the occipital lobe has decreased blood perfusion and neural activity, which was consistent with the view that visual spatial impairment was one of the main manifestations of T2DM (Cheung et al., 2010; Zeng et al., 2020). The preCG mainly manages the movement of skeletal muscles throughout the body (Li et al., 2015), which is called the motor area. Although the publication bias of this result leads to a decrease in the level of evidence, decreased motor and peripheral sensory abilities in T2DM patients with peripheral neuropathy may be due to a decrease in the neural impulses received by the preCG (Selvarajah et al., 2019), specifically manifested as a decrease in ALFF in T2DM. In addition, the CBF of T2DM reduced in IPG, which played an important role in the integration of human senses and the neural activity of determining the spatial position of objects, as well as in information processing in working memory (Koenigs et al., 2009). Working memory is an important process in brain cognition, especially in higher order cognition (Baddeley, 2003), and cognitive impairment in T2DM patients may be related to it. The regression analysis results of T2DM on MMSE also support this viewpoint.

We found that the ALFF of T2DM increased in CER and ITG.L. The cerebellar hemisphere is closely related to motor learning and coordination (Stoodley and Schmahmann, 2009), and the temporal lobe is related to memory, language fluency, language processing and language production, which are important components of cognitive ability (McCrimmon et al., 2012). Many studies have confirmed that T2DM patients would cause cognitive decline (McCrimmon et al., 2012; Biessels and Despa, 2018). Among the 11 T2DM studies we included in the analysis of ALFF, 6/11 were patients with MCI, 3/11 were patients with normal cognition, and 2/11 did not give cognitive assessment results. Therefore, we considered that the enhanced neural activity in these regions may play a role of compensation or supplement in T2DM patients, so that their cognitive performance can be retained or delayed to a certain extent. In addition, the CBF of T2DM increased in the SMA.R, which played an important role in precise control of motion, especially in fine movements such as finger movements (Tanji and Shima, 1994). Sensory and motor dysfunction caused by peripheral neuropathy complications in T2DM patients may be associated with an increase in CBF in this region (Allen et al., 2016).

This study showed that ALFF and CBF of PCUN, parietal lobe and occipital lobe in AD patients existed a consistent decline. The PCUN and parietal lobe are both the main brain regions that constitute the default pattern network (Bathelt and Geurts, 2021; Yeshurun et al., 2021), and are closely related to cognition (Smallwood et al., 2021). As a core brain region that affects visual spatial ability, the occipital lobe region also exhibits a covariate decrease in ALFF and CBF in AD patients, which may be related to perceptual impairments in visual and spatial abilities that are manifested early in AD patients (Mendez et al., 1990; Binetti et al., 1996; Zeng et al., 2020). In addition, studies have confirmed that normal visual ability had a significant impact on the development and persistence of cognitive ability. The parietal lobe and its adjacent occipital lobe are closely related to the temporal spatial structure function and graphic visual attention function (Sakkalou et al., 2021; Zhao et al., 2021). These changes in brain nerve activity and blood flow were closely related to the clinical manifestations of AD patients, such as acquired and persistent mental disorders, memory and cognitive dysfunction, speech and visual spatial skills disorders, and affected their social activities (Burns and Iliffe, 2009; Liu et al., 2019; Scheltens et al., 2021).

The results also showed that the ALFF of the CER.R, HIP.R and right striatum was higher in AD group than that in HCs, indicating that the neural activity in relevant brain regions was enhanced. The cerebellar hemisphere is closely related to motor learning (Stoodley and Schmahmann, 2009), HIP and striatum are important regions in the memory encoding pathway (Pennartz et al., 2011; Chersi and Burgess, 2015), and their anatomical relationship makes them more closely related. HIP and striatum can guide memory and behavior through cooperation or competition, and can regulate when other pathways in the brain are affected (Poldrack and Packard, 2003; Ghiglieri et al., 2011; Squire and Dede, 2015). The above brain regions are mainly related to learning and memory in cognitive activities. Due to the fact that memory impairment is the most significant clinical manifestation of AD patients (Burns and Iliffe, 2009), the above changes can be seen as a compensatory manifestation after memory related brain nerve activity damage.

After analyzing the brain regions with the same changes in T2DM patients and AD patients, the CBF of the two groups decreased uniformly only in the MOG.R region. As a key area of visual cortex, T2DM patients have visual space disorder and the occurrence of diabetes retinopathy also attributes to this change. The change of visual cortex in AD patients as a mediator, which further leaded to the impairment of advanced cognitive function, was also the focus of researchers.

After analyzing the neuroimaging evidence provided by the results of this study, we tend to believe that T2DM and AD are two diseases with their own characteristics of brain activity damage. The main damage area of T2DM was the bilateral occipital lobe, which mainly affects visual spatial function and other functions extended by visual function impairment. However, AD was mainly injured in bilateral PCUN and partial lobes, including posterior cingulate gyrus, PCUN, parietal lobe and part of occipital lobe, resulting in multi-dimensional functional damage in language, memory, learning, vision, etc. Only a small proportion (56 voxels in total) of MOG.R belonged to a part of the visual cortex were found in these two diseases, which was consistent with the clinical characteristics of them, and also suggested that T2DM was a risk factor for AD.

The reason why AD is considered as type 3 diabetes in some studies is briefly discussed tentatively. The main reason is that T2DM and AD have a high epidemiological correlation (Arvanitakis et al., 2004; Wang et al., 2012). However, as a high-risk factor for cerebrovascular diseases, T2DM can increase the risk of cerebral infarction and cerebral hemorrhage, which has achieved clinical consensus (Kannel and McGee, 1979). And as a complication of T2DM, cerebrovascular disease also has a higher incidence rate among T2DM patients (Gregg et al., 2016). Many clinical studies have shown that the occurrence of cerebrovascular events is significantly correlated with cognitive impairment and dementia (Vermeer et al., 2007; Troncoso et al., 2008; Rost et al., 2022). This correlation may help explain the epidemiological correlation between T2DM and AD (Sutherland et al., 2017). Researchers believe that another main reason why AD should be called type 3 diabetes is that T2DM and AD have many common pathophysiological bases, such as central insulin resistance (Janson et al., 2004; De Felice et al., 2022), AGEs and metabolic syndrome (Byun et al., 2017; Więckowska-Gacek et al., 2021). Firstly, lipid metabolism is an important component of metabolic syndrome. There is metabolic syndrome caused by insulin resistance in T2DM (Zheng et al., 2018), and autopsy findings of lipid particles in the brain of AD patients have also led researchers to suspect that lipid metabolism is involved in the pathogenesis of AD (Foley, 2010). In subsequent studies, it was found that sulfatides, an important subtype of sphingolipids, may play an important role in the pathogenesis of AD. Sulfatides are an important part of the myelin sheath and oligodendrocytes (Takahashi and Suzuki, 2012), and their consumption in AD patients is as high as 93% (Han et al., 2002). This change is a specific change of AD, but the pathogenesis of AD is more complex and still under study (Han, 2010; Cheng et al., 2013), and there is no clear evidence to confirm its correlation with abnormal lipid metabolism in diabetes. Secondly, in the past, there have been many studies on hyperglycemia leading to tissue damage through the production of AGEs, altering cell activation functions, and resulting in cognitive impairment (Klein and Waxman, 2003; Brownlee, 2005; Byun et al., 2017), but most of them are based on basic experiments (Batkulwar et al., 2018; Volpina et al., 2021). The impact of these findings on the human body is uncertain, and more evidence is needed to confirm whether this theory has a comorbidity pathway in T2DM and AD. Finally, there is increasing evidence that insulin resistance, especially central insulin resistance is related to the pathogenesis of AD (Janson et al., 2004; Neth and Craft, 2017; De Felice et al., 2022). Intranasal injection of insulin can alleviate memory deficits in some AD patients (Novak et al., 2014; Craft et al., 2017). However, the mechanism of insulin resistance on cognitive impairment in the brain is still unclear. The above results confirm that T2DM and AD are two closely related diseases, but it is still too early to call AD type 3 diabetes. In comparison, T2DM is a more appropriate high-risk factor for AD, and the relationship between the two diseases still needs further research.

## 5 Limitations

It should be noted that the following limitations still exist in this study. Firstly, all the literature was cross-sectional and lacked longitudinal tracking of disease progression. Secondly, this study conducted a meta-analysis based on the reported coordinates provided by the article or the corresponding author. Research results that do not provide coordinates are not included, which may cause bias. Thirdly, lack of sufficient data to correct the differences in data processing and the gray matter volume of subjects in the original study (Supplementary Tables S2, S3), which may potentially contribute to the high heterogeneity of our results. Fourthly, because most articles in the AD group did not provide the comorbidity of AD and T2DM, more detailed subgroup analysis cannot be performed. In the future, it is necessary to update the meta-analysis to eliminate the confounding factors of comorbidity and make the level of evidence higher. Fifthly, the population included in the study is mainly concentrated in the East Asian population, resulting in limited universality of the research results. Finally, it was hoped that in future studies, a larger sample of meta-analysis would be conducted, and attention will be paid to longitudinal studies from T2DM to T2DM with AD. Provide more core evidence for the occurrence and mechanism of comorbidity of the two diseases.

## 6 Conclusion

In summary, after analyzing the evidence provided by neuroimaging, T2DM and AD are two diseases with their own characteristics of brain neural activity and blood flow changes. Even if there is a small common area of reduced blood flow in both diseases, this is consistent with the clinical characteristics of both diseases and suggests a close relationship between the two diseases. This provided an idea for us to study the brain damage and the relationship between these two diseases in the future, and provided new insights for understanding the pathophysiology of brain changes in these two diseases and developing effective early intervention methods.

## Author contributions

HX: Data curation, Formal analysis, Investigation, Methodology, Software, Visualization, Writing – original draft, Writing – review & editing. YYu: Funding acquisition, Supervision, Validation, Software, Writing – original draft. YYa: Conceptualization, Methodology, Software, Validation, Writing – original draft. QS: Data curation, Validation, Writing – original draft. Z-YL: Conceptualization, Funding acquisition, Resources, Supervision, Validation, Writing – original draft. M-HN: Methodology, Project administration, Software, Writing – original draft. S-NL: Data curation, Software, Writing – original draft. PD: Data curation, Formal analysis, Writing – original draft. Y-YC: Software, Writing – original draft. X-YC: Software, Writing – original draft. NJ: Data curation, Methodology, Writing – original draft. L-JD: Supervision, Validation, Writing – original draft. WG: Data curation, Writing – original draft. J-JB: Software, Writing – original draft. L-FY: Resources, Software, Visualization, Writing – original draft, Writing – review & editing. G-BC: Conceptualization, Funding acquisition, Resources, Supervision, Validation, Writing – review & editing.

## Footnotes

^1^ https://www.crd.york.ac.uk/prospero/

^2^ https://www.nitrc.org/projects/bnv/

^3^ https://www.sdmproject.com/
