# Supplementary material for: Commonalities and distinctions between the type 2 diabetes mellitus and Alzheimer’s disease: a systematic review and multimodal neuroimaging meta-analysis
Source: Front Neurosci. 2023 Dec 6;17:1301778. doi: 10.3389/fnins.2023.1301778 (PMC10731270; doi:10.3389/fnins.2023.1301778)
Supplement: Supplementary file 1 [file Data_Sheet_1.docx]

**Supplementary Materials**

**Table S1. The detailed search strategy for the database**

| **Database** | **Search strategy** | |
| --- | --- | --- |
|  | **Part 1** | **Part 2** |
| **PubMed** | (((((((((((diabetes mellitus, type 2[MeSH Terms]) OR (Diabetes Mellitus, Type 2[Title/Abstract])) OR (Type 2 Diabetes[Title/Abstract])) OR (Diabetes Mellitus, Type II[Title/Abstract])) OR (NIDDM[Title/Abstract])) OR (T2DM[Title/Abstract])) OR (Alzheimer Disease[MeSH Terms])) OR (dementia[MeSH Terms])) OR (alzheimer*[Title/Abstract])) OR (dement*[Title/Abstract])) OR (AD[Title/Abstract])) AND ((((((amplitude of low frequency fluctuation[Title/Abstract]) OR (ALFF[Title/Abstract])) OR (low frequency fluctuation[Title/Abstract])) OR (LFF[Title/Abstract])) OR (amplitude of low frequency oscillation[Title/Abstract])) OR (LFO[Title/Abstract])) | (((((((((((diabetes mellitus, type 2[MeSH Terms]) OR (Diabetes Mellitus, Type 2[Title/Abstract])) OR (Type 2 Diabetes[Title/Abstract])) OR (Diabetes Mellitus, Type II[Title/Abstract])) OR (NIDDM[Title/Abstract])) OR (T2DM[Title/Abstract])) OR (Alzheimer Disease[MeSH Terms])) OR (dementia[MeSH Terms])) OR (alzheimer*[Title/Abstract])) OR (dement*[Title/Abstract])) OR (AD[Title/Abstract])) AND (((((Cerebrovascular Circulation[MeSH Terms]) OR (arterial spin labeling[Title/Abstract])) OR (ASL[Title/Abstract])) OR (Cerebral Blood Flow[Title/Abstract])) OR (CBF[Title/Abstract])) |
| **Web of science** | ((((((((((TS=(Diabetes Mellitus, Type 2)) OR TS=(Type 2 Diabetes)) OR TS=(Diabetes Mellitus, Type II)) OR TS=(NIDDM)) OR TS=(T2DM)) OR TS=(Alzheimer Disease)) OR TS=(dementia)) OR TS=(alzheimer*)) OR TS=(dement*)) OR TS=(AD)) and ((((((TS=(amplitude of low frequency fluctuation)) OR TS=(ALFF)) OR TS=(low frequency fluctuation)) OR TS=(LFF)) OR TS=(amplitude of low frequency oscillation)) OR TS=(LFO)) | ((((((((((TS=(Diabetes Mellitus, Type 2)) OR TS=(Type 2 Diabetes)) OR TS=(Diabetes Mellitus, Type II)) OR TS=(NIDDM)) OR TS=(T2DM)) OR TS=(Alzheimer Disease)) OR TS=(dementia)) OR TS=(alzheimer*)) OR TS=(dement*)) OR TS=(AD)) and (((((TS=(Cerebrovascular Circulation)) OR TS=(arterial spin labeling)) OR TS=(ASL)) OR TS=(Cerebral Blood Flow)) OR TS=(CBF)) |

**Table S2. Imaging characteristics of the ALFF studies included in the meta-analysis**

| **Study** | **Group** | **Scanner** | **Scan Duration** | **Software** | **Frequency range (Hz)** | **Smoothing kernel (mm)** | **Threshold (method)** | **GMV correction** | **Quality scores^a^** |
| --- | --- | --- | --- | --- | --- | --- | --- | --- | --- |
| Xia et al. | T2DM | 3,0 T | 12min20s | SPM8, DPARSF, REST | 0.01-0.08 | 4 | p＜0.05 (AlphaSim corrected) | NO | 20 |
| Chen et al. | T2DM | 3.0 T | 6min |  | 0.01-0.08 | 4 | p＜0.001 uncorrected | NO | 18 |
| Cui et al. | T2DM | 3,0 T | / | SPM8, DPARSF, REST | 0.01-0.08 | 4 | p＜0.005 (AlphaSim corrected) | NO | 17 |
| Wang et al. | T2DM | 3.0 T | 2min54s | SPM8, DPARSF, REST | 0.01-0.08 | 4 | p＜0.05 (AlphaSim corrected) | YES | 19 |
| Zhou et al. | T2DM | 3,0 T | 7min58s | SPM5, DPARSF, REST | 0.01-0.08 | 8 | p＜0.05 (Multiple Comparison corrected) | NO | 19 |
| Wang et al. | T2DM | 3.0 T | / | SPM8, DPARSF, REST | 0.01-0.08 | 8 | p＜0.05 (AlphaSim corrected) | NO | 18 |
| Yu et al. | T2DM | 3,0 T | ＞6min | SPM8, DPABI, REST | 0.01-0.08 | 8 | p＜0.05 (GRF corrected) | YES | 20 |
| Liu et al. | T2DM | 3.0 T | / | SPM8, DPABI | 0.01–0.1 | 4 | p＜0.05 (GRF corrected) | NO | 18 |
| Shi et al. | T2DM | 3,0 T | 15min | SPM, DPARSF, REST | 0.01-0.08 | / | P < 0.01 (AlphaSim corrected) | NO | 16 |
| Li et al. | T2DM | 3.0 T | / | SPM, REST | 0.01-0.08 | 6 | p＜0.05 (GRF corrected) | YES | 19 |
| Qi et al. | T2DM | 3.0 T | / | SPM8, DPABI | 0.01-0.08 | / | p＜0.05 (GRF corrected) | NO | 17 |
| Wang et al. | AD | 3.0 T | 7min58s | SPM5, DPARSF, REST | 0.01-0.08 | 4 | p＜0.05 (AlphaSim corrected) | NO | 19 |
| Xi et al. | AD | 1.5 T | / | SPM2, REST | 0.01-0.08 | 4 | p＜0.05 (Multiple Comparison corrected) | NO | 17 |
| Veldsman et al. | AD | 1.5 T | 8min | SPM8, REST | 0.01-0.08 | 8 | p＜0.05 (FDR corrected) | NO | 18 |
| Zheng et al. | AD | 3.0 T | 10min | SPM8, DPARSF | 0.01-0.08 | 4 | p＜0.05 (FDR corrected) | YES | 20 |
| Li et al. | AD | 1.5 T | 6min | SPM12, DPARSF | 0.01–0.1 | / | p＜0.05 (GRF corrected) | YES | 19 |
| Zeng et al. | AD | 3.0 T | 7min | SPM12, DPABI | 0.01-0.08 | 6 | p＜0.05 (GRF corrected) | YES | 19 |
| Zheng et al. | AD | 3.0 T | 6min | SPM12, DPARSF | 0.01-0.08 | 4 | p＜0.001 uncorrected | NO | 19 |
| Yang et al. | AD | 3.0 T | 7min58s | SPM12, GRETNA, REST | 0.01-0.08 | / | p＜0.05 (GRF corrected) | YES | 20 |
| Li et al. | AD | 3.0 T | ＞6min | SPM12, DPABI, | 0.01-0.08 | 6 | p＜0.005 (AlphaSim corrected) | NO | 19 |
| Chen et al. | AD | 3.0 T | 6min | SPM12, DPARSF, CAT12 | 0.01–0.08 | 6 | p＜0.05 (FDR corrected) | YES | 19 |
| Zhan et al. | AD | 3.0 T | 8min | SPM12, DAPRSF | 0.01–0.08 | 8 | p＜0.05 (FDR corrected) | NO | 19 |

GMV, gray matter volume; SPM, statistical parametric mapping; DPARSF, data processing assistant for resting-state fMRI; REST, the resting-state fMRI data analysis toolkit; DPABI, data processing and analysis for brain imaging; AFNI, analysis of functional neuroimages; FDR, false discovery rate; GRF, Gaussian random field.

^a^ Maximum score of 20 for each study.

/ means no relevant information was provided in the study.

**Table S3. Imaging characteristics of the CBF studies included in the meta-analysis**

| **Study** | **Scanner** | **Group** | **Scan**  **duration** | **Software** | **Labeling**  **Duration (ms)** | **Post label**  **Delay (ms)** | **Smoothing**  **Kernel (mm)** | **Threshold (method)** | **GMV correction** | **Quality scores^a^** |
| --- | --- | --- | --- | --- | --- | --- | --- | --- | --- | --- |
| Xia et al. | 3.0 T | T2DM | 45min | SPM8 | 600 | 1000 | 6 | P < 0.01 (FWE corrected) | YES | 18 |
| Jansen et al. | 3.0 T | T2DM | / | SPM8 | 1650 | 1525 | / | p < 0.05 (FDR corrected) | NO | 18 |
| Cui et al. | 3.0 T | T2DM | 8min | SPM8, AFNI | 600 | 1000 | 6 | P < 0.05 (AlphaSim corrected) | NO | 19 |
| Dai et al. | 3.0 T | T2DM | / | SPM8 | 1500 | 1500 | 8 | P < 0.05 (FWE corrected) | YES | 19 |
| Shen et al. | 3.0 T | T2DM | 6min17s | SPM8 | / | / | 6 | p < 0.001 uncorrected | NO | 17 |
| Zhang et al. | 3.0 T | T2DM | 8min31s | SPM8, REST | 1525 | 1525 | 8 | P < 0.01 (AlphaSim corrected) | YES | 19 |
| Huang et al. | 3.0 T | T2DM | / | SPM8, REST | 1525 | 1525 | 6 | p＜0.05 (GRF corrected) | NO | 16 |
| Yu et al. | 3.0 T | T2DM | ＞6min | SPM8, DPABI , REST | / | 2025 | 8 | p＜0.05 (GRF corrected) | YES | 20 |
| Asllani et al. | 1.5 T | AD | / | SPM99 | 2000 | 800 | 6 | p＜0.05 (Multiple Comparison corrected) | NO | 18 |
| Dai et al. | 1.5 T | AD | 7min44s | SPM2 | / | 700 | 6 | p＜0.05 (Multiple Comparison corrected) | NO | 18 |
| Yoshiura et al. | 3.0 T | AD | 5min20s | SPM2 | / | / | 12 | p < 0.001 uncorrected | NO | 16 |
| Chao et al. | 1.5 T | AD | 12min | SPM2 | / | / | 10 | p < 0.001 uncorrected | YES | 17 |
| Dashjamts et al. | 3.0 T | AD | 11min12s | SPM8 | / | / | 12 | p < 0.001 uncorrected | YES | 17 |
| Alexopoulos et al. | 3.0 T | AD | 7min18s | SPM5 | 700 | 800 | 12 | P < 0.05 (FWE corrected) | NO | 16 |
| Mak et al. | 3.0 T | AD | 6min | SPM5 | / | / | 8 | p < 0.001 uncorrected | YES | 16 |
| Kim et al. | 3.0 T | AD | / | SPM5 | 900 | 800 | 10 | p < 0.005 uncorrected | YES | 16 |
| Ding et al. | 3.0 T | AD | ＞5min | SPM8, AFNI | 1000 | 1500 | 6 | P < 0.05 (AlphaSim corrected) | NO | 20 |
| Roquet et al. | 3.0 T | AD | / | SPM8 | / | / | 8 | P < 0.05 (FWE corrected) | NO | 16 |
| Zheng et al. | 3.0 T | AD | / | SPM12, DAPRSF | 700 | 500 | 4 | P < 0.05 (FWE corrected) | NO | 19 |
| Duan et al. | 1.5 T | AD | / | SPM8 | 3700 | 700 | 6 | P < 0.05 (FDR corrected) | NO | 17 |
| Soman et al. | 3.0 T | AD | / | SPM12 | 1200 | 2025 | 8 | p < 0.05 (FDR corrected) | NO | 16 |

SPM, statistical parametric mapping; DPARSF, data processing assistant for resting-state fMRI; REST, the resting-state fMRI data analysis toolkit; DPABI, data processing and analysis for brain imaging; AFNI, analysis of functional neuroimages; FWE, family-wise error; FDR, false discovery rate; GRF, Gaussian random field.

^a^ Maximum score of 20 for each study.

/ means no relevant information was provided in the study.

**
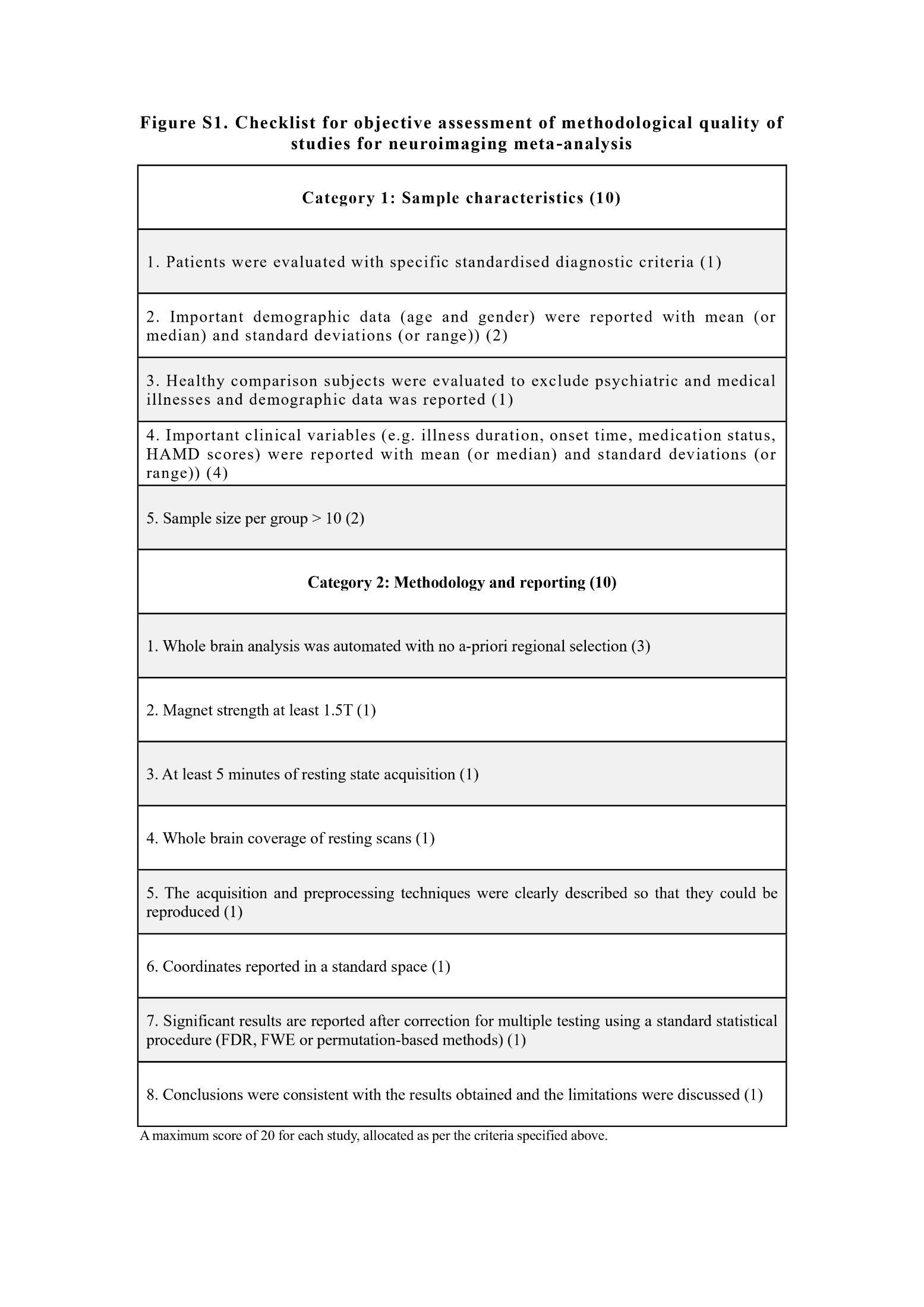
**

| **Table S4. Objective assessment of quality of all ALFF studies** | | | | | | | | | | | | | | | |  |  |  |  |
| --- | --- | --- | --- | --- | --- | --- | --- | --- | --- | --- | --- | --- | --- | --- | --- | --- | --- | --- | --- |
| **Study** | **Sample characteristics (10)** | | | | |  |  | | **Methodology and reporting (10)** | | | | | | | | |  | |
|  | standardised diagnostic criteria | Important demographic data (age and gender) | Healthy comparison subjects | Important clinical variables | Sample size per group > 10 |  | Whole brain analysis was automated | Magnet strength at least 1.5T | | At least 5 minutes of resting state acquisition | Whole brain coverage of resting scans | The acquisition and preprocessing techniques were clearly described | Coordinates reported in a standard space | FDR, FWE or permutation-based methods | Conclusions were consistent with the results | | **Quality scores** | |  |
| Xia et al. | **●** | **●●** | **●** | **●●●●** | **●●** |  | **●●●** | **●** | | **●** | **●** | **●** | **●** | **●** | **●** | | **20** | |  |
| Chen et al. | **●** | **●●** | **●** | **●●●○** | **●●** |  | **●●●** | **●** | | **●** | **●** | **●** | **●** | **○** | **●** | | **18** | |  |
| Cui et al. | **●** | **●●** | **●** | **●●●○** | **●●** |  | **●●●** | **●** | | **○** | **●** | **○** | **●** | **●** | **●** | | **17** | |  |
| Wang et al. | **●** | **●●** | **●** | **●●●●** | **●●** |  | **●●●** | **●** | | **○** | **●** | **●** | **●** | **●** | **●** | | **19** | |  |
| Zhou et al. | **○** | **●●** | **●** | **●●●●** | **●●** |  | **●●●** | **●** | | **●** | **●** | **●** | **●** | **●** | **●** | | **19** | |  |
| Wang et al. | **●** | **●●** | **●** | **●●●○** | **●●** |  | **●●●** | **●** | | **○** | **●** | **●** | **●** | **●** | **●** | | **18** | |  |
| Yu et al. | **●** | **●●** | **●** | **●●●●** | **●●** |  | **●●●** | **●** | | **●** | **●** | **●** | **●** | **●** | **●** | | **20** | |  |
| Liu et al. | **●** | **●●** | **●** | **●●●○** | **●●** |  | **●●○** | **●** | | **●** | **●** | **●** | **●** | **●** | **●** | | **18** | |  |
| Shi et al. | **●** | **●●** | **●** | **●●●○** | **●●** |  | **●●●** | **●** | | **●** | **●** | **●** | **●** | **●** | **●** | | **16** | |  |
| Li et al. | **●** | **●●** | **●** | **●●●●** | **●●** |  | **●●●** | **●** | | **○** | **●** | **●** | **●** | **●** | **●** | | **19** | |  |
| Qi et al. | **●** | **●●** | **●** | **●●○○** | **●●** |  | **●●●** | **●** | | **○** | **●** | **●** | **●** | **●** | **●** | | **17** | |  |
| Wang et al. | **●** | **●●** | **●** | **●●●○** | **●●** |  | **●●●** | **●** | | **●** | **●** | **●** | **●** | **●** | **●** | | **19** | |  |
| Xi et al. | **●** | **●●** | **●** | **●●●○** | **●●** |  | **●●●** | **●** | | **○** | **●** | **○** | **●** | **●** | **●** | | **17** | |  |
| Veldsman et al. | **●** | **●●** | **●** | **●●○○** | **●●** |  | **●●●** | **●** | | **●** | **●** | **●** | **●** | **●** | **●** | | **18** | |  |
| Zheng et al. | **●** | **●●** | **●** | **●●●●** | **●●** |  | **●●●** | **●** | | **●** | **●** | **●** | **●** | **●** | **●** | | **20** | |  |
| Li et al. | **●** | **●●** | **●** | **●●●○** | **●●** |  | **●●●** | **●** | | **●** | **●** | **●** | **●** | **●** | **●** | | **19** | |  |
| Zeng et al. | **●** | **●●** | **●** | **●●●○** | **●●** |  | **●●●** | **●** | | **●** | **●** | **●** | **●** | **●** | **●** | | **19** | |  |
| Zheng et al. | **●** | **●●** | **●** | **●●●●** | **●●** |  | **●●●** | **●** | | **●** | **●** | **●** | **●** | **○** | **●** | | **19** | |  |
| Yang et al. | **●** | **●●** | **●** | **●●●●** | **●●** |  | **●●●** | **●** | | **●** | **●** | **●** | **●** | **●** | **●** | | **20** | |  |
| Li et al. | **●** | **●●** | **●** | **●●●○** | **●●** |  | **●●●** | **●** | | **●** | **●** | **●** | **●** | **●** | **●** | | **19** | |  |
| Chen et al. | **●** | **●●** | **●** | **●●●○** | **●●** |  | **●●●** | **●** | | **●** | **●** | **●** | **●** | **●** | **●** | | **19** | |  |
| Zhan et al. | **●** | **●●** | **●** | **●●●○** | **●●** |  | **●●●** | **●** | | **●** | **●** | **●** | **●** | **●** | **●** | | **19** | |  |

**Table S5. Objective assessment of quality of all CBF studies**

| **Study** | **Sample characteristics** | | | | |  |  | | **Methodology and reporting** | | | | | | | |  | |
| --- | --- | --- | --- | --- | --- | --- | --- | --- | --- | --- | --- | --- | --- | --- | --- | --- | --- | --- |
|  | standardised diagnostic criteria | Important demographic data (age and gender) | Healthy comparison subjects | Important clinical variables | Sample size per group > 10 |  | Whole brain analysis was automated | Magnet strength at least 1.5T | | At least 5 minutes of resting state acquisition | Whole brain coverage of resting scans | The acquisition and preprocessing techniques were clearly described | Coordinates reported in a standard space | FDR, FWE or permutation-based methods | Conclusions were consistent with the results | **Quality scores** | |  |
| Xia et al. | ● | ●● | ● | ●●●○ | ●● |  | ●●○ | ● | | ● | ● | ● | ● | ● | ● | **18** | |  |
| Jansen et al. | ● | ●● | ● | ●●●○ | ●● |  | ●●● | ● | | ○ | ● | ● | ● | ● | ● | **18** | |  |
| Cui et al. | ● | ●● | ● | ●●●● | ●● |  | ●●○ | ● | | ● | ● | ● | ● | ● | ● | **19** | |  |
| Dai et al. | ● | ●● | ● | ●●●● | ●● |  | ●●● | ● | | ○ | ● | ● | ● | ● | ● | **19** | |  |
| Shen et al. | ● | ●● | ● | ●●○○ | ●● |  | ●●● | ● | | ● | ● | ● | ● | ○ | ● | **17** | |  |
| Zhang et al. | ● | ●● | ● | ●●●○ | ●● |  | ●●● | ● | | ● | ● | ● | ● | ● | ● | **19** | |  |
| Huang et al. | ● | ●● | ● | ●○○○ | ●● |  | ●●● | ○ | | ● | ● | ● | ● | ● | ● | **16** | |  |
| Yu et al. | ● | ●● | ● | ●●●● | ●● |  | ●●● | ● | | ● | ● | ● | ● | ● | ● | **20** | |  |
| Asllani et al. | ● | ●● | ● | ●●●○ | ●● |  | ●●● | ○ | | ● | ● | ● | ● | ● | ● | **18** | |  |
| Dai et al. | ● | ●● | ● | ●●○○ | ●● |  | ●●● | ● | | ● | ● | ● | ● | ● | ● | **18** | |  |
| Yoshiura et al. | ● | ●● | ● | ●○○○ | ●● |  | ●●● | ● | | ● | ● | ● | ● | ○ | ● | **16** | |  |
| Chao et al. | ● | ●● | ● | ●●○○ | ●● |  | ●●● | ● | | ● | ● | ● | ● | ○ | ● | **17** | |  |
| Dashjamts et al. | ● | ●● | ● | ●●○○ | ●● |  | ●●● | ● | | ● | ● | ● | ● | ○ | ● | **17** | |  |
| Alexopoulos et al. | ● | ●● | ● | ○○○○ | ●● |  | ●●● | ● | | ● | ● | ● | ● | ● | ● | **16** | |  |
| Mak et al. | ● | ●● | ● | ●○○○ | ●● |  | ●●● | ● | | ● | ● | ● | ● | ○ | ● | **16** | |  |
| Kim et al. | ● | ●● | ● | ●●○○ | ●● |  | ●●● | ○ | | ● | ● | ● | ● | ○ | ● | **16** | |  |
| Ding et al. | ● | ●● | ● | ●●●● | ●● |  | ●●● | ● | | ● | ● | ● | ● | ● | ● | **20** | |  |
| Roquet et al. | ● | ●● | ● | ●○○○ | ●● |  | ●●● | ○ | | ● | ● | ● | ● | ● | ● | **16** | |  |
| Zheng et al. | ● | ●● | ● | ●●●● | ●● |  | ●●● | ○ | | ● | ● | ● | ● | ● | ● | **19** | |  |
| Duan et al. | ● | ●● | ● | ●●●○ | ●● |  | ●●○ | ○ | | ● | ● | ● | ● | ● | ● | **17** | |  |
| Soman et al. | ● | ●● | ● | ●○○○ | ●● |  | ●●● | ○ | | ● | ● | ● | ● | ● | ● | **16** | |  |


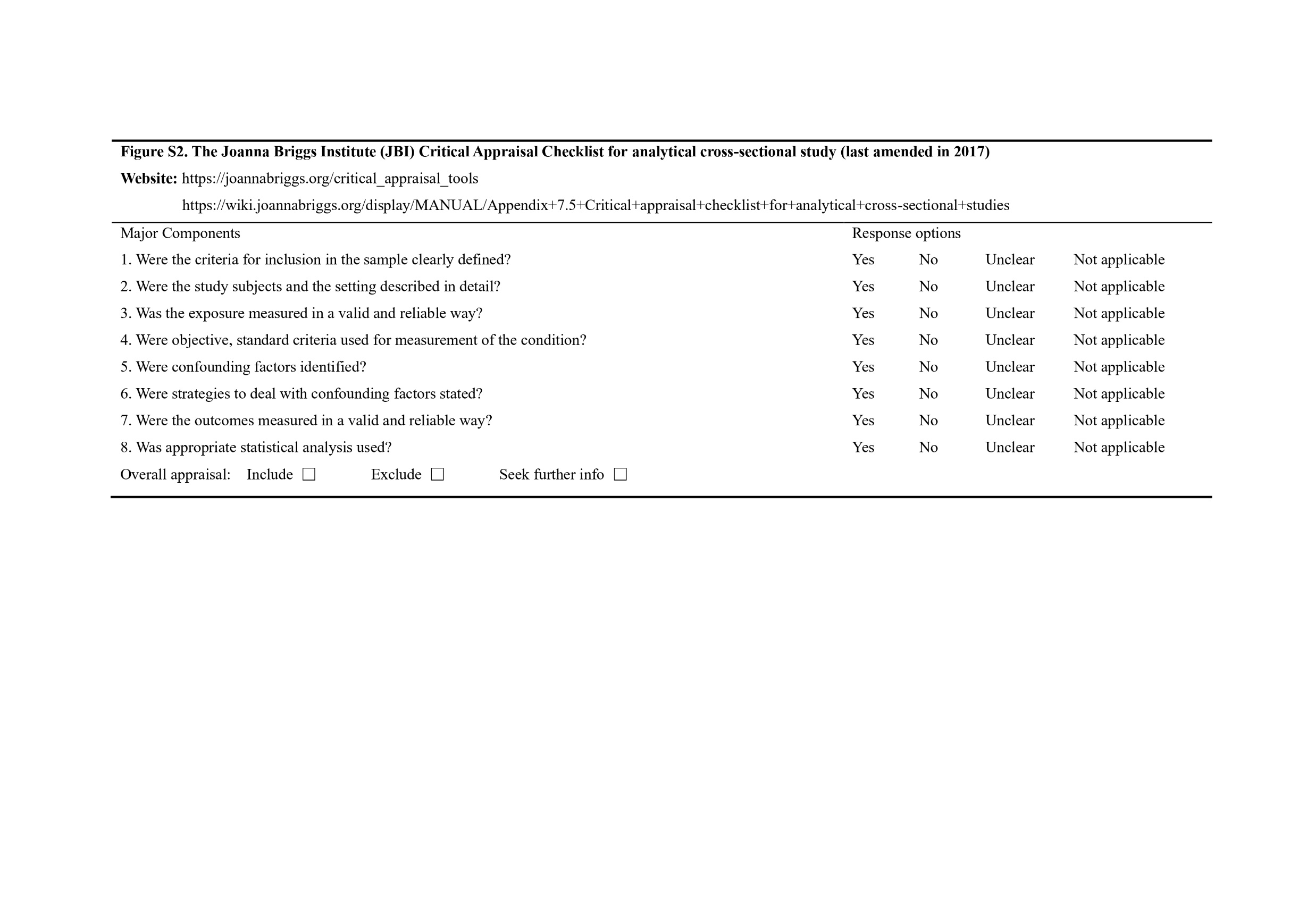


| **Table S6. Results of JBI scoring** | | | | | | |
| --- | --- | --- | --- | --- | --- | --- |
| **Analysis of ALFF** | | |  | **Analysis of CBF** | | |
| **Study** | **Group** | **JBI score** |  | **Study** | **Group** | **JBI score** |
| Xia et al. (2013) | T2DM | 16 |  | Xia et al. (2015) | T2DM | 14 |
| Chen et al. (2014) | T2DM | 14 |  | Jansen et al. (2016) | T2DM | 14 |
| Cui et al. (2014) | T2DM | 13 |  | Cui et al. (2017) | T2DM | 15 |
| Wang et al. (2014) | T2DM | 13 |  | Dai et al. (2017) | T2DM | 14 |
| Zhou et al. (2014) | T2DM | 14 |  | Shen et al. (2017) | T2DM | 13 |
| Wang et al. (2017) | T2DM | 15 |  | Zhang et al. (2019) | T2DM | 14 |
| Yu et al. (2019) | T2DM | 16 |  | Huang et al. (2021) | T2DM | 12 |
| Liu et al. (2020) | T2DM | 14 |  | Yu et al. (2019) | T2DM | 16 |
| Shi et al. (2020) | T2DM | 12 |  | Asllani et al. (2008) | AD | 14 |
| Li et al. (2021) | T2DM | 14 |  | Dai et al. (2009) | AD | 14 |
| Qi et al. (2020) | T2DM | 13 |  | Yoshiura et al. (2009) | AD | 12 |
| Wang et al. (2011) | AD | 15 |  | Chao et al. (2010) | AD | 13 |
| Xi et al. (2012) | AD | 13 |  | Dashjamts et al. (2011) | AD | 13 |
| Veldsman et al. (2017) | AD | 14 |  | Alexopoulos et al. (2012) | AD | 12 |
| Zheng et al. (2018) | AD | 16 |  | Mak et al. (2012) | AD | 12 |
| Li et al. (2019) | AD | 14 |  | Kim et al. (2013) | AD | 12 |
| Zeng et al. (2019) | AD | 14 |  | Ding et al. (2014) | AD | 16 |
| Zheng et al. (2019) | AD | 15 |  | Roquet et al. (2016) | AD | 14 |
| Yang et al. (2020) | AD | 16 |  | Zheng et al. (2019) | AD | 15 |
| Li et al. (2021) | AD | 14 |  | Duan et al. (2020) | AD | 12 |
| Chen et al. (2022) | AD | 14 |  | Soman et al. (2021) | AD | 12 |
| Zhan et al. (2022) | AD | 14 |  |  |  |  |

JBI score greater than 70% of the total score was considered as low risk

| **Table S7. Meta-analyses results regarding ALFF difference between T2DM and HCs** | | | | | |  |  |  |
| --- | --- | --- | --- | --- | --- | --- | --- | --- |
| **Local maximum region** | **Peak MNI**  **coordinate (x, y, z)** | **SDM**  **Z value** | **p value** | **Cluster**  **(NO. of voxels)** | **Breakdown (No. of voxels)** | **Egger’s test**  **(p value)** | **Heterogeneity (I^2^)** | **Jackknife sensitivity** |
| **T2DM vs HCs (T2DM＞HCs)** | | | | | |  |  |  |
| Right cerebellum, crus II | 22, -74, -42 | 1.975 | 3.61E-05 | 1885 | Right cerebellum, hemispheric lobule VIII (957) | 0.998 | ＞50% | 11/11 |
|  |  |  |  |  | Right cerebellum, crus II (318) |  |  |  |
|  |  |  |  |  | Right cerebellum, hemispheric lobule VIIB (231) |  |  |  |
|  |  |  |  |  | Right cerebellum, hemispheric lobule IX (50) |  |  |  |
| Left cerebellum, hemispheric lobule VIII | -34, -46, -50 | 1.798 | 1.60E-04 | 559 | Left cerebellum, hemispheric lobule VIII (383) | 0.926 | ＞50% | 11/11 |
|  |  |  |  |  | Left cerebellum, hemispheric lobule VIIB (106) |  |  |  |
| Left inferior temporal gyrus, BA 20 | -52, -10, -30 | 1.717 | 2.79E-04 | 419 | Left inferior temporal gyrus, BA 20 (221) | 0.805 | ＞50% | 10/11 |
|  |  |  |  |  | Left inferior network, inferior longitudinal fasciculus (77) |  |  |  |
| **T2DM vs HCs (T2DM＜HCs)** | | | | | |  |  |  |
| Left calcarine fissure / surrounding cortex, BA 17 | -14, -100, -2 | -1.613 | 7.69E-04 | 733 | Left middle occipital gyrus, BA 18 (256) | 0.734 | ＞50% | 10/11 |
|  |  |  |  |  | Left middle occipital gyrus, BA 17 (137) |  |  |  |
|  |  |  |  |  | Left inferior network, inferior longitudinal fasciculus (69) |  |  |  |
|  |  |  |  |  | Left optic radiations (60) |  |  |  |
|  |  |  |  |  | Left inferior occipital gyrus, BA 18 (60) |  |  |  |
|  |  |  |  |  | Corpus callosum (55) |  |  |  |
| Right inferior occipital gyrus, BA 18 | 26, -90, -10 | -1.935 | 1.03E-05 | 302 | Right lingual gyrus, BA 18 (84) | 0.601 | ＞50% | 11/11 |
|  |  |  |  |  | Right inferior occipital gyrus, BA 18 (65) |  |  |  |
|  |  |  |  |  | Right inferior occipital gyrus, BA 19 (55) |  |  |  |
| Left precentral gyrus, BA 6 | -30, -6, 54 | -1.496 | 1.76E-03 | 189 | Left precentral gyrus, BA 6 (96) | ＜0.05 | ＞50% | 9/11 |

T2DM, type 2 diabetes mellitus, HCs, health controls, ALFF, amplitude of low frequency fluctuation.

| **Table S8. Meta-analyses results regarding ALFF difference between AD and HCs** | | | | | |  |  |  |
| --- | --- | --- | --- | --- | --- | --- | --- | --- |
| **Local maximum region** | **Peak MNI**  **coordinate (x, y, z)** | **SDM**  **Z value** | **p value** | **Cluster**  **(NO. of voxels)** | **Breakdown (No. of voxels)** | **Egger’s test**  **(p value)** | **Heterogeneity (I^2^)** | **Jackknife sensitivity** |
| **AD vs HCs (AD＞HCs)** | | | | | | | | |
| Right cerebellum, hemispheric lobule VIIB | 38, -46, -42 | 1.666 | 3.56E-04 | 506 | Right cerebellum, crus I (136) | 0.348 | ＞50% | 11/11 |
|  |  |  |  |  | Right cerebellum, crus II (85) |  |  |  |
|  |  |  |  |  | Right cerebellum, hemispheric lobule VIII (54) |  |  |  |
| (undefined) | 2, 18, -14 | 1.36 | 2.12E-03 | 368 | Right striatum (86) | 0.333 | ＞50% | 10/11 |
| Right hippocampus, BA 34 | 20, -6, -14 | 1.496 | 1.15E-03 | 316 | Right cortico-spinal projections (57) | 0.705 | ＞50% | 10/11 |
|  |  |  |  |  | Right amygdala, BA 34 (50) |  |  |  |
| **AD vs HCs (AD＜HCs)** | | | | | | | | |
| Right precuneus | 8, -68, 30 | -2.101 | ~0 | 2202 | Left precuneus (287) | 0.918 | ＞50% | 11/11 |
|  |  |  |  |  | Right precuneus (261) |  |  |  |
|  |  |  |  |  | Right median network, cingulum (203) |  |  |  |
|  |  |  |  |  | Right precuneus, BA 23 (181) |  |  |  |
|  |  |  |  |  | Left precuneus, BA 7 (171) |  |  |  |
|  |  |  |  |  | Right precuneus, BA 7 (105) |  |  |  |
|  |  |  |  |  | Right cuneus cortex (101) |  |  |  |
|  |  |  |  |  | Corpus callosum (96) |  |  |  |
|  |  |  |  |  | Left precuneus, BA 23 (87) |  |  |  |
|  |  |  |  |  | Right cuneus cortex, BA 19 (84) |  |  |  |
|  |  |  |  |  | Left posterior cingulate gyrus, BA 23 (78) |  |  |  |
|  |  |  |  |  | Right median cingulate / paracingulate gyri, BA 23 (75) |  |  |  |
| Right superior temporal gyrus, BA 42 | 60, -30, 16 | -1.436 | 8.83E-04 | 416 | Right superior temporal gyrus, BA 42 (160) | 0.969 | ＞50% | 11/11 |
|  |  |  |  |  | Right superior temporal gyrus, BA 22 (82) |  |  |  |

AD, Alzheimer's disease, HCs, health controls, ALFF amplitude of low frequency fluctuation.

| **Table S9. Meta-analyses results regarding CBF difference between T2DM and HCs** | | | | | |  |  |  |
| --- | --- | --- | --- | --- | --- | --- | --- | --- |
| **Local maximum region** | **Peak MNI**  **coordinate (x, y, z)** | **SDM**  **Z value** | **p value** | **Cluster**  **(NO. of voxels)** | **Breakdown**  **(No. of voxels)** | **Egger’s test**  **(p value)** | **Heterogeneity (I^2^)** | **Jackknife sensitivity** |
| **T2DM vs HCs (T2DM＞HCs)** | | | | | | | |  |
| Right supplementary motor area, BA 6 | 6, -12, 68 | 1.243 | 7.48E-04 | 299 | Right supplementary motor area, BA 6 (172) | 0.335 | ＞50% | 7/8 |
| **T2DM vs HCs (T2DM＜HCs)** | | | | | | | |  |
| Left optic radiations | -18, -86, 4 | -2.016 | 3.61E-05 | 995 | Left middle occipital gyrus, BA 18 (196) | 0.833 | ＞50% | 8/8 |
|  |  |  |  |  | Corpus callosum (177) |  |  |  |
|  |  |  |  |  | Left inferior network, inferior longitudinal fasciculus (124) |  |  |  |
|  |  |  |  |  | Left optic radiations (103) |  |  |  |
|  |  |  |  |  | Left middle occipital gyrus, BA 17 (103) |  |  |  |
|  |  |  |  |  | Left calcarine fissure / surrounding cortex, BA 17 (93) |  |  |  |
|  |  |  |  |  | Left inferior occipital gyrus, BA 18 (82) |  |  |  |
| Left calcarine fissure / surrounding cortex, BA 18 | 4, -88, 8 | -1.600 | 5.47E-04 | 1079 | Right inferior network, inferior longitudinal fasciculus (276) | 0.673 | ＞50% | 8/8 |
|  |  |  |  |  | Right middle occipital gyrus, BA 39 (170) |  |  |  |
|  |  |  |  |  | Right calcarine fissure / surrounding cortex, BA 17 (109) |  |  |  |
|  |  |  |  |  | Right calcarine fissure / surrounding cortex, BA 18 (108) |  |  |  |
|  |  |  |  |  | Corpus callosum (98) |  |  |  |
|  |  |  |  |  | Right middle occipital gyrus, BA 19 (94) |  |  |  |
| Right inferior parietal gyri, BA 40 | 40, -42, 48 | -1.521 | 1.18E-03 | 161 | Right inferior parietal gyri, BA 40 (85) | 0.934 | ＞50% | 8/8 |

T2DM, type 2 diabetes mellitus, HCs, health controls, CBF cerebral blood flow.

| **Table S10. Meta-analyses results regarding CBF difference between AD and HCs** | | | | | |  |  |  |
| --- | --- | --- | --- | --- | --- | --- | --- | --- |
| **Local maximum region** | **Peak MNI**  **coordinate (x, y, z)** | **SDM**  **Z value** | **p value** | **Cluster**  **(NO. of voxels)** | **Breakdown (No. of voxels)** | **Egger’s test**  **(p value)** | **Heterogeneity (I^2^)** | **Jackknife sensitivity** |
| **AD vs HCs (AD＜HCs)** | | | | | | | |  |
| Left precuneus | 0, -62, 34 | -4.925 | ~0 | 5318 | Left precuneus (591) | 0.955 | ＞50% | 13/13 |
|  |  |  |  |  | Right precuneus (512) |  |  |  |
|  |  |  |  |  | Left median network, cingulum (474) |  |  |  |
|  |  |  |  |  | Left median cingulate / paracingulate gyri (415) |  |  |  |
|  |  |  |  |  | Right median network, cingulum (383) |  |  |  |
|  |  |  |  |  | Right median cingulate / paracingulate gyri (352) |  |  |  |
|  |  |  |  |  | Corpus callosum (344) |  |  |  |
|  |  |  |  |  | Right median cingulate / paracingulate gyri, BA 23 (326) |  |  |  |
|  |  |  |  |  | Left median cingulate / paracingulate gyri, BA 23 (298) |  |  |  |
|  |  |  |  |  | Left precuneus, BA 7 (255) |  |  |  |
|  |  |  |  |  | Right precuneus, BA 7 (163) |  |  |  |
|  |  |  |  |  | Left posterior cingulate gyrus, BA 23 (135) |  |  |  |
|  |  |  |  |  | Right precuneus, BA 23 (131) |  |  |  |
|  |  |  |  |  | Left precuneus, BA 23 (130) |  |  |  |
| Left inferior parietal gyri, BA 40 | -46, -56, 42 | -4.121 | ~0 | 2233 | Left inferior parietal gyri, BA 40 (866) | 0.777 | ＞50% | 13/13 |
|  |  |  |  |  | Left angular gyrus, BA 39 (426) |  |  |  |
|  |  |  |  |  | Left inferior parietal gyri, BA 7 (200) |  |  |  |
|  |  |  |  |  | Left superior parietal gyrus, BA 7 (183) |  |  |  |
|  |  |  |  |  | Left inferior parietal gyri, BA 39 (91) |  |  |  |
|  |  |  |  |  | Left superior longitudinal fasciculus II (76) |  |  |  |
|  |  |  |  |  | Left angular gyrus, BA 7 (65) |  |  |  |
| Right inferior parietal gyri, BA 39 | 50, -56, 42 | -2.871 | 3.1E-05 | 1357 | Right angular gyrus, BA 39 (397) | 0.960 | ＞50% | 13/13 |
|  |  |  |  |  | Right inferior parietal gyri, BA 40 (354) |  |  |  |
|  |  |  |  |  | Right supramarginal gyrus, BA 40 (157) |  |  |  |
|  |  |  |  |  | Right angular gyrus, BA 7 (110) |  |  |  |
|  |  |  |  |  | Right middle occipital gyrus, BA 39 (85) |  |  |  |

AD, Alzheimer's disease, HCs, health controls, CBF cerebral blood flow.

| **Table S11. Sensitivity analysis of ALFF in T2DM** | | | | | | |
| --- | --- | --- | --- | --- | --- | --- |
| **Study** | **CER.R** | **CER.L** | **ITG.L** | **MOG.L** | **IOG.R** | **preCG.L** |
| Xia et al. (2013) | **√** | **√** | **√** | **√** | **√** | **√** |
| Chen et al. (2014) | **√** | **√** | **√** | **√** | **√** | **√** |
| Cui et al. (2014) | **√** | **√** | **√** | **√** | **√** | **√** |
| Wang et al. (2014) | **√** | **√** | **×** | **√** | **√** | **√** |
| Zhou et al. (2014) | **√** | **√** | **√** | **√** | **√** | **×** |
| Wang et al. (2017) | **√** | **√** | **√** | **√** | **√** | **√** |
| Yu et al. (2019) | **√** | **√** | **√** | **√** | **√** | **√** |
| Liu et al. (2020) | **√** | **√** | **√** | **√** | **√** | **√** |
| Shi et al. (2020) | **√** | **√** | **√** | **√** | **√** | **×** |
| Li et al. (2021) | **√** | **√** | **√** | **√** | **√** | **√** |
| Qi et al. (2020) | **√** | **√** | **√** | **×** | **√** | **√** |
| **Total** | **11 out of 11** | **11 out of 11** | **10 out of 11** | **10 out of 11** | **11 out of 11** | **9 out of 11** |

CER.R, right cerebellum, CER.L, left cerebellum, ITG.L, left inferior temporal gyrus, MOG.L, left middle occipital gyrus, IOG.R, right inferior occipital gyrus, preCG.L, left precentral gyrus.

| **Table S12. Sensitivity analysis of CBF in T2DM** |  |  |  |  |  |
| --- | --- | --- | --- | --- | --- |
| **Study** | **SMA.R** | **MOG.L** | **MOG.R** | **IPG.L** | **IPG.R** |
| Xia et al. (2015) | **√** | **√** | **√** | **×** | **√** |
| Jansen et al. (2016) | **√** | **√** | **√** | **√** | **√** |
| Cui et al. (2017) | **√** | **√** | **√** | **√** | **√** |
| Dai et al. (2017) | **√** | **√** | **√** | **√** | **√** |
| Shen et al. (2017) | **√** | **√** | **√** | **√** | **√** |
| Zhang et al. (2019) | **×** | **√** | **√** | **√** | **√** |
| Huang et al. (2021) | **√** | **√** | **√** | **√** | **√** |
| Yu et al. (2019) | **√** | **√** | **√** | **√** | **√** |
| **Total** | **7 out of 8** | **8 out of 8** | **8 out of 8** | **7 out of 8** | **8 out of 8** |

SMA.R, right supplementary motor area, MOG.L, left middle occipital gyrus, MOG.R, right middle occipital gyrus, IPG.L, left inferior parietal gyri, IPG.R, right inferior parietal gyri.

| **Table S13. Sensitivity analysis of ALFF in AD** | | | | | | |
| --- | --- | --- | --- | --- | --- | --- |
| **Study** | **CER.R** | **HIP.R** | **striatum.R** | **STG.R** | **preCG.L** | **preCG.R** |
| Wang et al. (2011) | **√** | **√** | **√** | **√** | **√** | **√** |
| Xi et al. (2012) | **√** | **√** | **√** | **√** | **√** | **√** |
| Veldsman et al. (2017) | **√** | **√** | **√** | **√** | **√** | **√** |
| Zheng et al. (2018) | **√** | **√** | **√** | **√** | **√** | **√** |
| Li et al. (2019) | **√** | **√** | **√** | **√** | **√** | **√** |
| Zeng et al. (2019) | **√** | **√** | **√** | **√** | **√** | **√** |
| Zheng et al. (2019) | **√** | **√** | **√** | **√** | **√** | **√** |
| Yang et al. (2020) | **√** | **√** | **√** | **√** | **√** | **√** |
| Li et al. (2021) | **√** | **√** | **√** | **√** | **√** | **√** |
| Chen et al. (2022) | **√** | **×** | **×** | **√** | **√** | **√** |
| Zhan et al. (2022) | **√** | **√** | **√** | **√** | **√** | **√** |
| **Total** | **11 out of 11** | **10 out of 11** | **10 out of 11** | **11 out of 11** | **11 out of 11** | **11 out of 11** |

CER.R, right cerebellum, HIP.R, right hippocampus, striatum.R, right striatum, STG.R, right superior temporal gyrus, preCG.L, left precuneus, preCG.R, right precuneus.

| **Table S14. Sensitivity analysis of CBF in AD** | | | | |
| --- | --- | --- | --- | --- |
| **Study** | **IPG.L** | **IPG.R** | **PCUN.L** | **PCUN.R** |
| Asllani et al. (2008) | **√** | **√** | **√** | **√** |
| Dai et al. (2009) | **√** | **√** | **√** | **√** |
| Yoshiura et al. (2009) | **√** | **√** | **√** | **√** |
| Chao et al. (2010) | **√** | **√** | **√** | **√** |
| Dashjamts et al. (2011) | **√** | **√** | **√** | **√** |
| Alexopoulos et al. (2012) | **√** | **√** | **√** | **√** |
| Mak et al. (2012) | **√** | **√** | **√** | **√** |
| Kim et al. (2013) | **√** | **√** | **√** | **√** |
| Ding et al. (2014) | **√** | **√** | **√** | **√** |
| Roquet et al. (2016) | **√** | **√** | **√** | **√** |
| Zheng et al. (2019) | **√** | **√** | **√** | **√** |
| Duan et al. (2020) | **√** | **√** | **√** | **√** |
| Soman et al. (2021) | **√** | **√** | **√** | **√** |
| **Total** | **13 out of 13** | **13 out of 13** | **13 out of 13** | **13 out of 13** |

IPG.L, left inferior parietal gyri, IPG.R, right inferior parietal gyri, PCUN.L, left precuneus, PCUN.R, right precuneus.

| **Table S15. Analyses of heterogeneity and publication bias** | | | | | | | |
| --- | --- | --- | --- | --- | --- | --- | --- |
| **Peak MNI coordinate (x, y, z)** | **Bias** | **z value** | **df** | **p value** | **TES value** | **Heterogeneity(I^2^)** | **Subgroup** |
| **ALFF abnormalities in T2DM** | | | | | |  |  |
| 22, -74, -42 | -0.01 | 0.00 | 9 | 0.998 | 0.691 | ＞50% |  |
| -34, -46, -50 | -0.27 | -0.09 | 9 | 0.926 | 0.407 | ＞50% |  |
| -52, -10, -30 | 0.79 | 0.25 | 9 | 0.805 | 0.298 | ＞50% |  |
| -14, -100, -2 | 1.05 | 0.34 | 9 | 0.734 | 0.962 | ＞50% |  |
| 26, -90, -10 | 1.58 | 0.52 | 9 | 0.601 | 1.000 | ＞50% |  |
| -30, -6, 54 | -6.99 | -2.56 | 9 | ＜0.05 | 0.000 | ＞50% |  |
| **ALFF abnormalities in AD** | | | | | |  |  |
| 38, -46, -42 | -1.55 | -0.94 | 9 | 0.348 | 0.995 | ＞50% |  |
| 2, 18, -14 | 1.65 | 0.97 | 9 | 0.333 | 0.970 | ＞50% |  |
| 20, -6, -14 | 0.55 | 0.38 | 9 | 0.705 | 0.298 | ＞50% |  |
| 8, -68, 30 | -0.15 | -0.10 | 9 | 0.918 | 0.000 | ＞50% |  |
| 60, -30, 16 | 0.06 | 0.04 | 9 | 0.969 | 0.970 | ＞50% |  |
| **CBF abnormalities in T2DM** | | | | | |  |  |
| 6, -12, 68 | 6.06 | 0.96 | 6 | 0.335 | 0.885 | ＞50% |  |
| -18, -86, 4 | 1.51 | 0.21 | 6 | 0.833 | 0.690 | ＞50% |  |
| 4, -88, 8 | 3.02 | 0.42 | 6 | 0.673 | 0.850 | ＞50% |  |
| 40, -42, 48 | -0.60 | -0.08 | 6 | 0.934 | 1.000 | ＞50% |  |
| **CBF abnormalities in AD** | | | | | |  |  |
| 0, -62, 34 | -0.09 | -0.06 | 11 | 0.955 | 0.998 | ＞50% |  |
| -46, -56, 42 | 0.48 | 0.28 | 11 | 0.777 | 0.995 | ＞50% |  |
| 50, -56, 42 | -0.09 | -0.05 | 11 | 0.960 | 0.000 | ＞50% |  |
| **Both ALFF and CBF abnormalities in AD** | | | | | |  |  |
| 6, -64, 30 | -1.48 | -0.96 | 9 | 0.339 | 0.651 | ＞50% | ALFF |
|  | 0.82 | 0.47 | 11 | 0.640 | 0.595 | ＞50% | CBF |
| **Both ALFF and CBF abnormalities in T2DM** | | | | | |  |  |
| 24, -92, 8 | 1.91 | 0.61 | 9 | 0.545 | 1.000 | ＞50% | ALFF |
|  | 3.52 | 0.50 | 6 | 0.614 | 0.839 | ＞50% | CBF |
| -20, -88, 2 | 0.46 | 0.14 | 9 | 0.887 | 0.999 | ＞50% | ALFF |
|  | 1.84 | 0.21 | 6 | 0.833 | 0.700 | ＞50% | CBF |
| **CBF abnormalities in both T2DM and AD** | | | | | |  |  |
| 44, -74, 26 | 2.31 | 0.31 | 6 | 0.754 | 0.824 | ＞50% | T2DM |
|  | -0.67 | -0.33 | 11 | 0.745 | 0.000 | ＞50% | AD |

TES, test of excess significance
